# Endophilin-A2-dependent tubular endocytosis promotes plasma membrane repair and parasite invasion

**DOI:** 10.1242/jcs.249524

**Published:** 2020-12-01

**Authors:** Matthias Corrotte, Mark Cerasoli, Fernando Y. Maeda, Norma W. Andrews

**Affiliations:** 1 Department of Cell Biology and Molecular Genetics, University of Maryland, College Park, MD 20742, USA; 2 Department of Veterinary Medicine, VA-MD College of Veterinary Medicine, University of Maryland, College Park, MD 20742, USA

**Keywords:** Endocytosis, Endophilin, Plasma membrane repair

## Abstract

Endocytosis of caveolae has previously been implicated in the repair of plasma membrane wounds. Here, we show that caveolin-1-deficient fibroblasts lacking caveolae upregulate a tubular endocytic pathway and have a reduced capacity to reseal after permeabilization with pore-forming toxins compared with wild-type cells. Silencing endophilin-A2 expression inhibited fission of endocytic tubules and further reduced plasma membrane repair in cells lacking caveolin-1, supporting a role for tubular endocytosis as an alternative pathway for the removal of membrane lesions. Endophilin-A2 was visualized in association with cholera toxin B-containing endosomes and was recruited to recently formed intracellular vacuoles containing *Trypanosoma cruzi*, a parasite that utilizes the plasma membrane wounding repair pathway to invade host cells. Endophilin-A2 deficiency inhibited *T. cruzi* invasion, and fibroblasts deficient in both caveolin-1 and endophilin-A2 did not survive prolonged exposure to the parasites. These findings reveal a novel crosstalk between caveolin-1 and endophilin-A2 in the regulation of clathrin-independent endocytosis and plasma membrane repair, a process that is subverted by *T. cruzi* parasites for cell invasion.

## Abstract (precis)

**Summary:** Crosstalk between endophilin-A2 and caveolin-1, proteins implicated in clathrin-independent endocytosis, regulates a tubular endocytic pathway important for plasma membrane repair and trypanosome invasion.

## INTRODUCTION

For injured cells to survive, plasma membrane (PM) wounds must be rapidly repaired. After several decades of investigation, it is now clear that eukaryotic cells do not reseal spontaneously. Wound repair is fast and critically dependent on the influx of extracellular Ca^2+^, which triggers a sequence of steps culminating in the restoration of PM integrity (Andrews and Corrotte, 2018). Among the better defined roles of Ca^2+^ influx through PM wounds are the recruitment of annexins (Swaggart et al., 2014), activation of the muscle-specific Ca^2+^-binding protein dysferlin (Bansal et al., 2003) and exocytosis of lysosomes at sites of PM injury (Cheng et al., 2014; Forestier et al., 2011; Ibata et al., 2019; Luisoni et al., 2015; Reddy et al., 2001). Ca^2+^-dependent exocytosis was initially proposed to be sufficient to promote cell resealing, by patching the wound (Terasaki et al., 1997) or reducing PM tension (Togo et al., 2000). Subsequent studies revealed that exocytosis of lysosomes is followed by a fast, clathrin-independent form of endocytosis (Idone et al., 2008b; Miller et al., 2015). This surprising finding gave rise to the concept that PM repair involves sequential membrane remodeling steps that include exocytosis of lysosomes, followed by lesion removal through endocytosis (Idone et al., 2008a; Keefe et al., 2005; McDade et al., 2014).

The ability of conventional lysosomes to undergo Ca^2+^-regulated exocytosis was first identified during studies of the mechanism by which the protozoan parasite *Trypanosoma cruzi* invades host cells (Tardieux et al., 1992). Trypomastigotes, the *T. cruzi* infective stages, are motile cells of 10–15 µm in length that can invade and replicate intracellularly in a large number of nucleated cell types. The large size of these parasites and their ability to form tight membrane-bound parasitophorous vacuoles initially suggested that host cell invasion might involve actin-dependent phagocytosis (Burleigh and Andrews, 1995). Surprisingly, host cell actin polymerization proved dispensable for *T. cruzi* invasion, suggesting that these parasites utilize a distinct, ubiquitous cellular machinery to gain access to the intracellular environment (Sibley and Andrews, 2000). Detailed examination of this process revealed that *T. cruzi* trypomastigotes enter host cells by a highly unusual mechanism – by triggering Ca^2+^ signaling and exocytosis of lysosomes at sites of parasite attachment, followed by the formation of parasitophorous vacuoles that contain both early endosomal and lysosomal markers (Andrade and Andrews, 2004; Fernandes et al., 2011; Tardieux et al., 1992). Strikingly, marked similarities were identified between the mechanisms mediating host cell invasion by *T. cruzi* and the repair of PM wounds. *T. cruzi* trypomastigotes transiently wound the host cell PM, triggering release of lysosomal hydrolases that stimulate endocytosis and promote the formation of unique, ceramide-enriched parasitophorous vacuoles. These studies indicate that *T. cruzi* parasites hijack the lysosome and endocytosis-mediated PM repair mechanism for host cell invasion (Fernandes et al., 2011).

Caveolae are morphologically homogeneous PM invaginations of less than 100 nm found in many cell types. Two groups of cytosolic scaffolding proteins, caveolins and cavins, are required for the assembly of caveolae on PM microdomains that are enriched in lipid-raft markers such as cholesterol and sphingolipids (Parton and Simons, 2007). Caveolae are particularly abundant in cells susceptible to mechanical stress, such as muscle fibers and endothelial cells, and there is evidence that flattening of caveolae helps protect the PM from mechanical damage (Sinha et al., 2011). An investigation of the mechanism by which mammalian cells reseal after attack by the pore-forming toxin streptolysin O (SLO) revealed that toxin pores can be internalized within caveolar vesicles (Corrotte et al., 2013) and trafficked to lysosomes for degradation (Corrotte et al., 2012). Notably, RNAi-mediated silencing of caveolin-1 (Cav1) expression inhibits PM resealing in cells permeabilized by pore-forming toxins and also by mechanical scraping, suggesting that caveolar endocytosis (Pelkmans and Helenius, 2002) is a form of clathrin-independent endocytosis that mediates the repair of different forms of PM injury (Andrews et al., 2014; Corrotte et al., 2013).

B lymphocytes, which do not form morphologically distinct caveolae (Fra et al., 1994), also reseal after injury with SLO by a process involving lysosomal exocytosis followed by endocytosis (Miller et al., 2015). Interestingly, SLO-permeabilized B cells upregulate a tubular endocytic pathway (Miller et al., 2015), raising the possibility that, when proteins necessary for the assembly of caveolae are absent, lipid raft PM microdomains may be mobilized for internalization in the form of larger tubule-shaped endosomes. In this study we have extended our investigation of PM repair in caveolae-deficient cells by examining mouse embryonic fibroblasts (MEFs) derived from Cav1 knockout (KO) mice, in parallel with MEFs from wild-type (WT) littermates. Our results revealed that in the absence of Cav1, the Bin-Amphiphysin-Rvs (BAR) domain-containing protein endophilin-A2 (EndoA2) assumes a central role in regulating a tubular endocytic pathway that promotes PM repair. Consistent with the extensive functional similarities previously identified between PM repair and *T. cruzi* invasion, we show that recruitment of EndoA2 to tubular PM invaginations plays a critical role in the mechanism by which the intracellular protozoan parasite *T. cruzi* invades host cells.

## RESULTS

### Cav1 knockout MEFs have reduced PM repair capacity

**Fig. 1.**
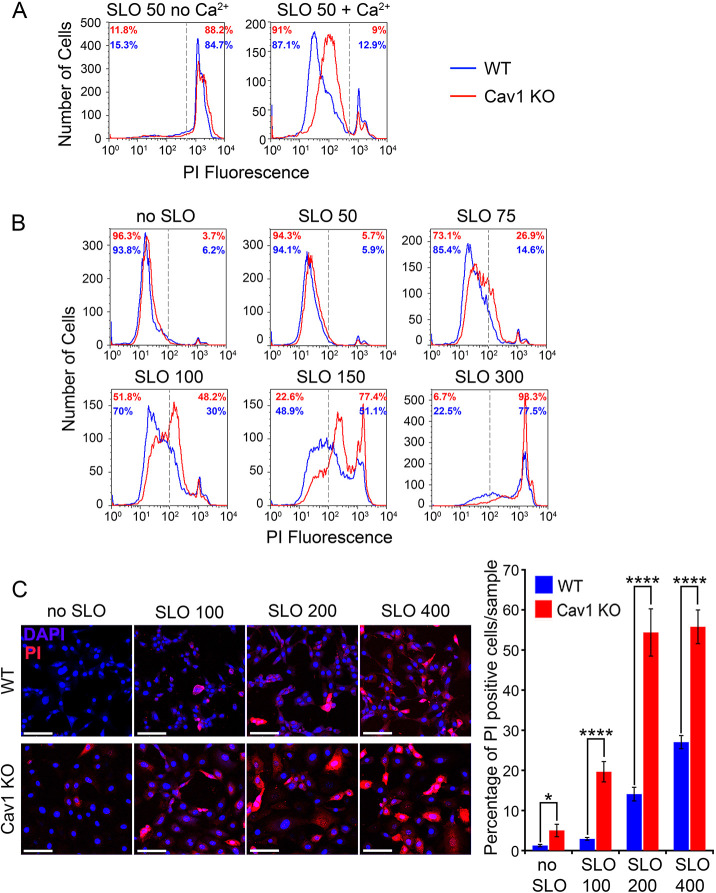
**Cav1 knockout MEFs have a reduced PM repair capacity.** (A) Flow cytometry of WT (blue) and Cav1 KO MEFs (red) exposed to 50 ng/ml SLO in the presence or absence of Ca^2+^ at 37°C for 5 min and stained with propidium iodide (PI) to detect permeabilized cells. The results are representative of three independent experiments. (B) Flow cytometry of WT (blue) and Cav1 KO MEFs (red) exposed to increasing concentrations of SLO (50–300 ng/ml) in the presence of Ca^2+^ at 37°C for 1 min and stained with PI. The results are representative of four independent experiments. In A and B the vertical dashed lines represent the gate used to calculate cell population percentages. (C) Fluorescence microscopy images of attached WT and Cav1 KO MEFs treated with increasing concentrations of SLO (ng/ml) for 1 min in the presence of Ca^2+^ and stained with DAPI (blue) and PI (red). Scale bars: 50 µm. The graph shows the mean±s.e.m. percentage of PI-positive cells determined for each condition in three independent experiments. *P=0.04; ****P<0.0001 (unpaired two-tailed Student's *t*-test).

To further investigate the mechanism of caveolae-independent PM repair detected in B cells (Miller et al., 2015), we used MEFs derived from WT and Cav1 KO littermate mice (Razani et al., 2001) and performed 5 min SLO wounding assays, followed by staining with the membrane-impermeable dye propidium iodide (PI) and flow cytometry analysis to assess the extent of PM repair (Idone et al., 2008b). In the absence of Ca^2+^, a condition that does not allow PM repair, WT and Cav1 KO MEFs were equally susceptible to permeabilization with 50 ng/ml SLO, as indicated by the similar percentages of cells with high PI staining (88.2% for Cav1 KO and 84.7% for WT; Fig. 1A, no Ca^2+^). In the presence of Ca^2+^, a condition permissive for PM repair, the high-PI cell populations were markedly reduced (9% for Cav1 KO and 12.9% for WT), indicating that both cell types were able to remove SLO pores, blocking entry of the membrane-impermeable dye (Fig. 1A, + Ca^2+^). However, a rightward shift in the low-PI population fluorescence levels was observed in Cav1 KO MEFs (Fig. 1A, + Ca^2+^, red line), suggesting that these cells were not as efficient as WT MEFs (Fig. 1A, blue line) in blocking PI entry after SLO permeabilization.

To further investigate the apparently reduced PM repair capacity of Cav1 KO MEFs, we shortened the assay time to 1 min and performed SLO dose-dependent wounding repair assays in the presence of Ca^2+^ (Fig. 1B). As expected, the ability of MEFs to reseal their PM decreased as the amount of SLO increased. Interestingly, after permeabilization with SLO concentrations above 75 ng/ml, increasingly higher levels of PI staining were detected in Cav1 KO than in WT cells. This difference was measured by placing the fluorescence quantification gate next to the PI-negative populations not exposed to SLO (Fig. 1B). Notably, at 100 and 150 ng/ml SLO, large populations of Cav1 KO cells with intermediate levels of fluorescence intensity were observed, suggesting that some influx of PI was occurring under those conditions, possibly through a few SLO pores that remained on the PM. Both cell populations showed a larger resealing defect after exposure to 300 ng/ml SLO, but even under these high-wounding conditions 22.5% of WT MEFs blocked PI entry, compared to only 6.7% in Cav1 KO MEFs. We also performed SLO permeabilization and repair assays in attached cells, and the percentage of PI-positive cells was significantly higher in Cav1 KO MEFs when compared to WT MEFs (Fig. 1C). Overall, these results confirm that both Cav1 KO and WT cells can repair SLO-induced PM wounds, but that Cav1 KO MEFs are less efficient.

### Cholera toxin B is taken up predominantly through caveolae-like vesicles in WT MEFs and through tubular endosomes in Cav1 KO MEFs

**Fig. 2.**
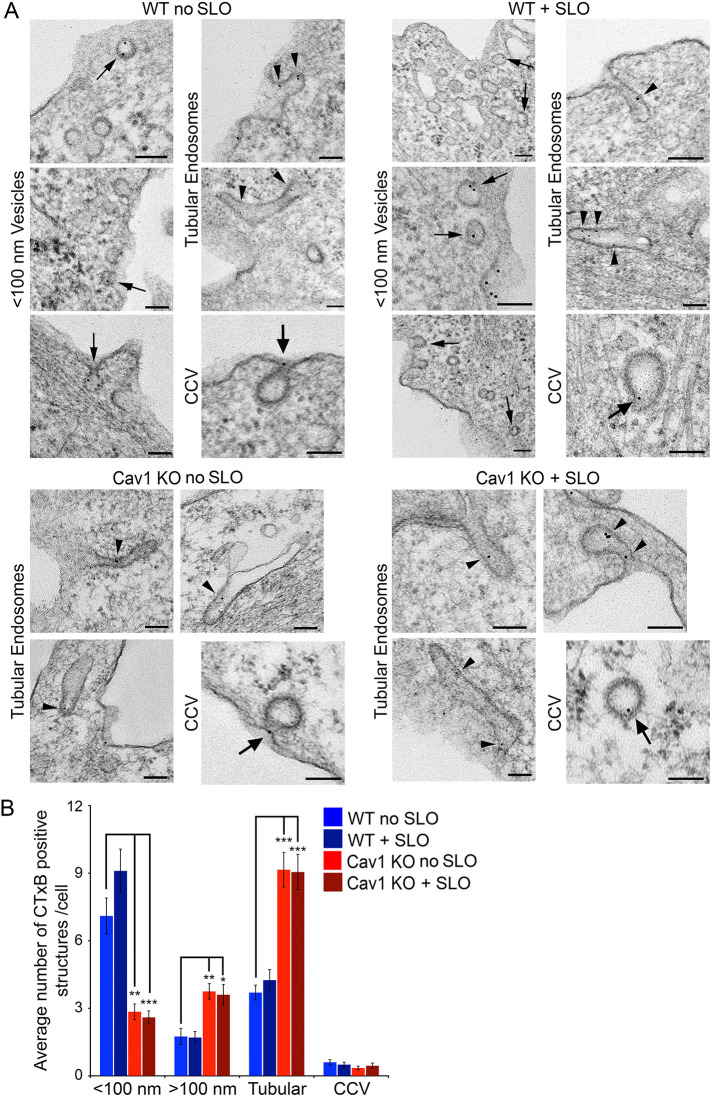
**CTxB endocytosis occurs predominantly through caveolae-like small vesicles in WT MEFs but shifts to tubular endosomes in Cav1 KO MEFS.** (A) TEM images of WT and Cav1 KO MEFs treated or not with SLO in the presence of CTxB–gold for 1 min at 37°C. Small arrows: <100 nm vesicles containing CTxB–gold. Arrowheads: tubular endosomes. Large arrows: CCVs. Scale bars: 100 nm. (B) Quantification of <100 nm vesicles, >100 nm vesicles, tubular endosomes and CCVs containing CTxB–gold in WT and Cav1 KO MEFs treated as in A. All vesicles containing CTxB–gold were counted in 20 individual cell sections/sample. The data represent the mean±s.e.m. of CTxB–gold-containing vesicles/cell section and are representative of two independent experiments. *P=0.029; **P<0.01; ***P<0.001 (unpaired two-tailed Student's *t*-test comparing each sample with the WT no SLO control condition).

To investigate whether the differences observed in the resealing capacity of SLO-permeabilized WT or Cav1 KO MEFs were associated with changes in endocytic carriers, we used transmission electron microscopy (TEM) to examine recently formed intracellular compartments containing cholera toxin B (CTxB), which binds to the GM1 glycosphingolipid that is concentrated on lipid rafts and caveolae (Parton, 1994; Pelkmans and Helenius, 2002). CTxB is a well-established cargo of clathrin-independent endocytosis (Boucrot et al., 2015; Renard et al., 2015), but can also enter cells through additional endocytic pathways (Stoeber et al., 2012). Cells were treated or not with SLO for 1 min in the presence of CTxB–gold conjugates, and after fixation and TEM processing, membrane-bound compartments containing the tracer were quantified on randomly-acquired images (Fig. 2A). Four categories of endocytic compartments containing CTxB were identified: caveolae-like vesicles smaller than 100 nm (<100 nm), vesicles larger than 100 nm (>100 nm), tubular endosomes, and clathrin-coated vesicles (CCV). Consistent with previous reports (Parton, 1994), in WT MEFs, CTxB was most frequently detected within uncoated caveolae-like vesicles smaller than 100 nm. Some CTxB was found in uncoated tubular endosomes, whereas markedly lower amounts were present in larger vesicles and in CCVs (Fig. 2A,B, WT). Thus, although CTxB can enter cells through various endocytic routes (Ewers and Helenius, 2011; Torgersen et al., 2001), in the MEFs used in this study, caveolae-like vesicles smaller than 100 nm are the preferred pathway for endocytosis after a short 1 min incubation period.

Strikingly, a marked change was observed in the morphology of CTxB endocytic carriers in Cav1 KO MEFs. In these cells that lack caveolae, most of the tracer was present inside elongated tubular compartments, often visualized in continuity with the PM (Fig. 2A, Cav1 KO, arrowheads). Consistent with the lack of morphologically distinct caveolae, only very few vesicles smaller than 100 nm containing CTxB were found in Cav1 KO MEFs, with or without SLO treatment. This decrease in the number of caveolae-like vesicles in Cav1 KO MEFs was accompanied by a dramatic increase in tubular endocytic compartments containing CTxB, whereas only a very small fraction of the CtxB was detected in CCVs (Fig. 2A,B, Cav1 KO). These results suggest that Cav1 deficiency in MEFs is associated with upregulation of a population of clathrin-independent tubular endosomes, perhaps as a compensatory mechanism for the absence of caveolae, as previously observed in B lymphocytes (Miller et al., 2015).

### EndoA2 is required for the scission of tubular endosomes containing CTxB and for PM repair in Cav1 KO MEFs

**Fig. 3.**
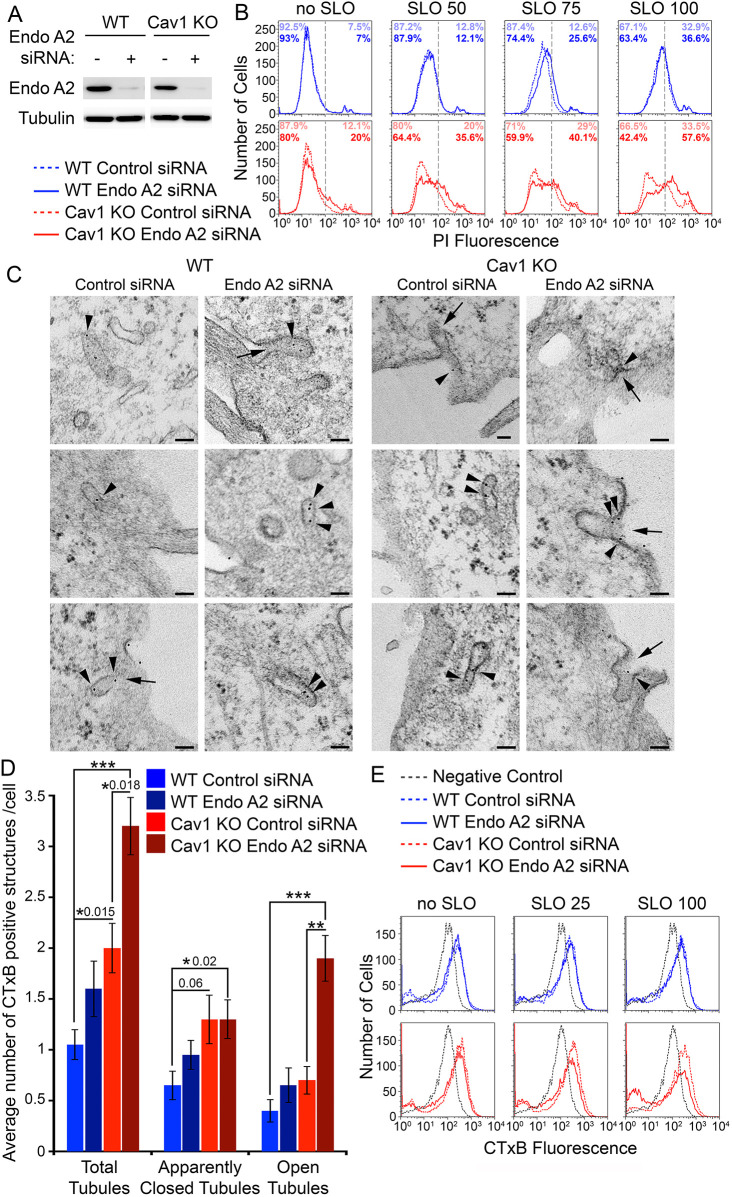
**EndoA2 promotes fission of tubular endosomes and PM repair in Cav1 KO MEFs.** (A) Western blot using antibodies against EndoA2 and tubulin (loading control) of WT and Cav1 KO MEF lysates treated with control (−) or EndoA2 (+) siRNA. (B) Flow cytometry of WT (blue) and Cav1 KO MEFs (red) treated with control (dashed lines) or EndoA2 (full lines) siRNA for 48 h, exposed to increasing concentrations of SLO (50–100 ng/ml) in the presence of Ca^2+^ at 37°C for 2 min, and stained with PI to detect permeabilized cells. The vertical dashed lines represent the gate utilized to calculate cell population percentages (light color, control siRNA; dark color, EndoA2 siRNA). The results are representative of six independent experiments. (C) TEM images of tubular endosomes in WT and Cav1 KO MEFs treated with control or EndoA2 siRNA for 48 h and incubated with CTxB–gold for 1 min at 37°C. Arrowheads: CTxB–gold. Arrows: open tubular endosomes. Scale bars: 100 nm. (D) Quantification of total, apparently closed and open tubular endosomes containing CTxB–gold in WT and Cav1 KO MEFs treated as in C. All tubular endosomes containing CTxB–gold were counted in 20 individual cell sections/sample. The data represent the mean±s.e.m. of CTxB–gold-containing tubular endosomes/cell section, and are representative of two independent experiments. *P=0.015–0.02; **P<0.01; ***P<0.001 (unpaired two-tailed Student's *t*-test comparing each sample with the WT control siRNA condition). (E) Flow cytometry of WT (blue) and Cav1 KO MEFs (red) treated with control (dashed lines) or EndoA2 (full lines) siRNA for 48 h and exposed to SLO (25 and 100 ng/ml) or not (no SLO) in the presence of Alexa Fluor 488–CTxB for 10 min at 37°C, followed by quenching with anti-Alexa Fluor 488 antibodies and Trypan Blue for 20 min at 4°C. Negative controls (gray dashed lines) represent WT or Cav1 KO MEFs incubated with Alexa Fluor 488–CTxB at 4°C followed by quenching at 4°C, without stimulating endocytosis at 37°C. The results are representative of three independent experiments.

Next, we investigated the role in PM repair of the tubular endocytic pathway upregulated in the absence of caveolae. To this end, we used siRNA in both WT and Cav1 KO MEFs to inhibit the expression of proteins previously reported to participate in tubular endocytosis, such as galectin-3 (Lakshminarayan et al., 2014) and endophilins (Ambroso et al., 2014; Renard et al., 2015). After confirming that siRNA treatment decreased expression of these proteins (Fig. 3A; Fig. S1B,D,F), we determined the ability of WT and Cav1 KO MEFs to reseal their PM after permeabilization with SLO for 2 min. RNAi-mediated knockdown of galectin-3, endophilin-A1 and endophilin-A3 had no effect on the typical PI-staining pattern of each cell type in the flow cytometry assay, reflecting their PM repair capacity (Fig. S1A,C,E). We also tested the involvement of clathrin-dependent endocytosis by reducing levels of clathrin heavy chain expression, which inhibited endocytosis of biotinylated surface proteins but had no impact on PM repair (data not shown).

On the other hand, RNAi-mediated knockdown of EndoA2 (Fig. 3A) significantly increased the number of PI-positive Cav1 KO cells when compared to controls, accentuating the PM repair defect observed in these cells. In contrast, no consistent inhibition of PM repair was observed in WT MEFs after reducing EndoA2 expression levels (Fig. 3B). This result suggested that EndoA2 might be required for PM repair only in Cav1 KO MEFs, possibly as a consequence of the observed shift from caveolar to tubular endocytosis (Fig. 2B). To investigate this possibility, we used TEM to examine CTxB-carrying tubular endocytic structures generated within 1 min in WT and Cav1 KO MEFs that had been previously treated or not with EndoA2 siRNA. In agreement with the results discussed above (Fig. 2B), significantly more CTxB-containing tubular endosomes were observed in Cav1 KO MEFs when compared to WT MEFs. Notably, treatment with EndoA2 siRNA significantly increased the number of CTxB-containing tubular endosomes visualized in Cav1 KO MEFs, but not in WT MEFs (Fig. 3D, total tubules). Importantly, EndoA2-deficient Cav1 KO MEFs contained a significantly larger number of tubular endosomes that were ‘open’, in direct continuity with the PM, when compared to those in WT MEFs and Cav1 KO cells treated with control siRNA (Fig. 3C, arrows; Fig. 3D, open tubules). In both WT and Cav1 KO MEFs, the numbers of CTxB-containing tubular endosomes not visualized in direct continuity with the PM (Fig. 3D, apparently closed tubules) were similar in cells treated or not with EndoA2 siRNA, suggesting that open tubules accounted for the overall increase in the total number of CTxB-carrying tubular endosomes observed in EndoA2-depleted Cav1 KO MEFs (Fig. 3D). Thus, inhibition of EndoA2 expression appeared to either increase the formation of tubular endosomes or to inhibit their fission from the PM, resulting in the accumulation of open tubular endocytic compartments at the PM.

To distinguish between these two possible scenarios, we incubated WT and Cav1 KO MEFs with Alexa Fluor 488–CTxB for 10 min, followed by quenching of the extracellular fluorescence and detection of the internalized toxin by flow cytometry. The levels of CTxB endocytosis in WT MEFs were similar in cells treated with either control or EndoA2 siRNA, with or without SLO wounding (Fig. 3E, WT). This result indicates that EndoA2 expression does not influence CTxB endocytosis in WT MEFs, in agreement with the relatively low numbers of CTxB-positive tubular endosomes observed in this cell type (Fig. 2B). However, in Cav1 KO MEFs, EndoA2 siRNA treatment inhibited CTxB endocytosis when compared to that in cells treated with control siRNA (Fig. 3E, Cav1 KO). This decrease was accentuated in a dose-dependent manner by exposure to SLO, suggesting that an EndoA2-dependent form of endocytosis is specifically mobilized when Cav1 KO MEFs are injured with SLO. Taken together with the inhibition of PM repair caused by EndoA2 depletion specifically in Cav1 KO MEFs (Fig. 3B), these results suggest that Cav1 KO cells upregulate a tubular endocytic pathway that requires EndoA2 for fission from the PM, a function necessary for the removal of lesions and cell resealing.

**Fig. 4.**
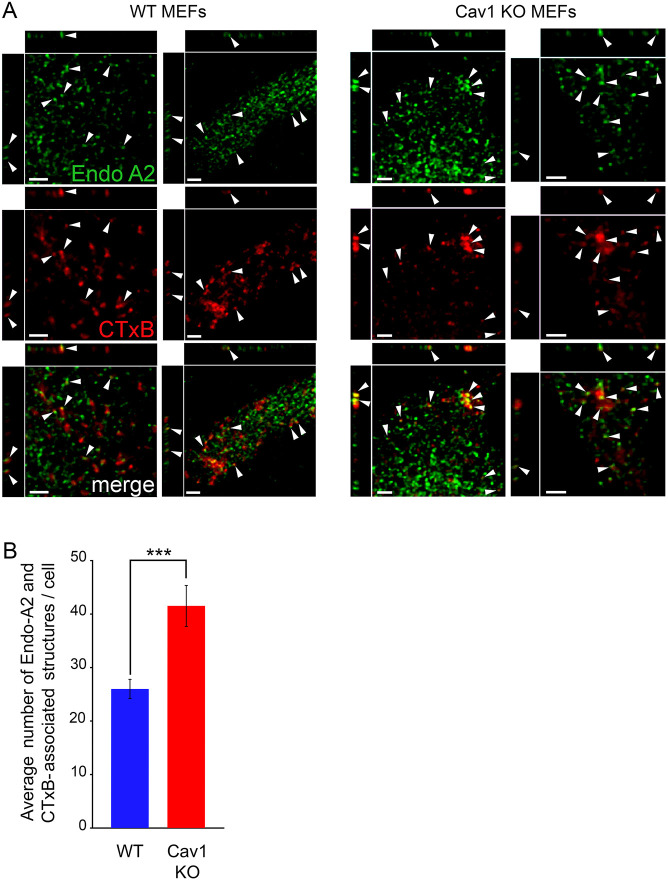
**EndoA2 partially colocalizes with endocytosed CTxB in WT and Cav1 KO MEFs.** (A) Confocal fluorescence microscopy images of WT and Cav1 KO MEFs incubated with fluorescent CTxB (red) for 15 s at 37°C and stained with anti-EndoA2 antibodies (green). The arrowheads point to EndoA2 puncta that partially colocalize with CTxB. Scale bars: 2 µm. The images are representative of three independent experiments. (B) Quantification of juxtaposed EndoA2 and CTxB puncta. The data represent the mean±s.e.m. of juxtaposition events detected in >20 cells, in three independent experiments. ***P<0.001 (unpaired two-tailed Student's *t*-test).

To examine the interaction of EndoA2 with cargo internalized during the brief periods of time involved in PM repair (Idone et al., 2008b; Steinhardt et al., 1994), we performed immunofluorescence assays using antibodies against EndoA2 in cells incubated with fluorescent CTxB for 15 s. In both WT and Cav1 KO MEFs, endogenous EndoA2 was detected in a punctate pattern on the ventral surface of cells,_­_ as previously reported (Boucrot et al., 2015). In WT MEFs, a few EndoA2 and CTxB puncta appeared juxtaposed, in a ‘strawberry’ pattern where CTxB puncta were capped by EndoA2 (Fig. 4A, arrowheads). Cav1 KO MEFs, however, had significantly higher levels of these partially colocalized CTxB–EndoA2 structures (Fig. 4A,B). In light of the data discussed above and previous reports (Boucrot et al., 2015), these immunofluorescence results suggest that the juxtaposed CTxB–EndoA2 structures that were more frequently detected in Cav1 KO MEFs may correspond to EndoA2-dependent tubular endosomes.

### EndoA2 promotes cell invasion by *Trypanosoma cruzi* and is recruited to nascent parasite-containing intracellular vacuoles

**Fig. 5.**
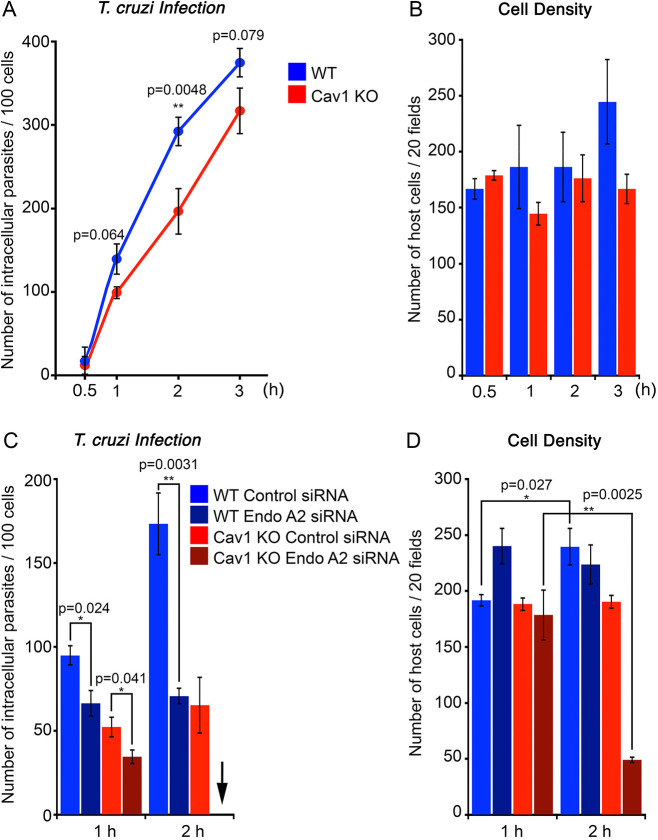
**Cell invasion by *T. cruzi* is inhibited by EndoA2 depletion, and leads to host cell death when combined with Cav1 deficiency.** (A–D) Cells were incubated with *T. cruzi* for various periods of time, fixed and stained with anti-*T. cruzi* antibodies and DAPI to quantify intracellular parasites. (A) Intracellular *T. cruzi* in WT (blue) or Cav1 KO (red) MEFs over time, expressed as intracellular parasites/100 cells. The data represents the mean±s.e.m. of triplicates. **P<0.01 (unpaired Student's *t*-tests comparing WT and Cav1 KO samples). (B) Cell density in *T. cruzi*-infected cultures, as in A. DAPI-stained cells in 20 microscopic fields (1000× magnification) were counted. The data represents the mean±s.e.m. of triplicates. Unpaired, two-tailed Student's *t*-tests were performed comparing each value with the corresponding 0.5 h time point; *P*-values were not significant (>0.05). (C) Intracellular *T. cruzi* after 1 or 2 h of exposure to WT (blue) or Cav1 KO (red) MEFs pre-treated with control (light color) or EndoA2 siRNA (dark color). The data represent the mean±s.e.m. of triplicate quantification of intracellular parasites in 100 cells. The arrow indicates the condition (EndoA2 siRNA, 2 h infection) where invasion was not quantified due to cell loss. *P<0.05; **P<0.01 (unpaired Student's *t*-test). (D) Cell density in *T. cruzi*-infected cultures, as in C. DAPI-stained cells in 20 microscopic fields (1000× magnification) were counted. The data represent the mean±s.e.m. of triplicates. *P<0.05; **P<0.01 (unpaired Student's *t*-test).

Previous studies have shown that the intracellular protozoan *T. cruzi* wounds the PM of host cells, triggering a repair process that is subverted by the parasites for invasion (Fernandes et al., 2011). Given that several interventions that inhibit PM repair also inhibit *T. cruzi* invasion (Chakrabarti et al., 2003; Fernandes et al., 2011; Fernandes et al., 2007; Reddy et al., 2001), we examined the impact of EndoA2 depletion on the susceptibility of WT and Cav1 KO MEFs to infection by these parasites. We first exposed both cell types to infective stages of *T. cruzi* for increasing periods of time and found that Cav1 KO MEFs were slightly less susceptible than WT MEFs to infection (Fig. 5A). Both cell types were able to repair *T. cruzi*-induced PM damage, because no host cell loss was observed over an assay period of 3 h (Fig. 5B). However, when siRNA was used to silence EndoA2 expression, a significant reduction in invasion was observed in both WT and Cav1 KO MEFs after 1 h of exposure to the parasites (Fig. 5C, 1 h). During this period, there was also no significant cell loss in MEF cultures treated with control or EndoA2 siRNA (Fig. 5D, 1 h). After a 2 h infection period, a marked reduction in parasite invasion was again observed in WT MEFs treated with EndoA2 siRNA, and host cells were not lost from cultures treated with control or EndoA2 siRNA (Fig. 5D, WT 2 h). In contrast, treatment with EndoA2 siRNA followed by 2 h of exposure to *T. cruzi* resulted in marked host cell loss from Cav1 KO cultures, preventing the quantification of parasite invasion at this time point (Fig. 5D, Cav1 KO 2 h). Thus, whereas MEFs lacking Cav1 are partially less susceptible than WT MEFs to *T. cruzi* infection, deficiency in both Cav1 and EndoA2 strongly impairs the cells' ability to survive during the parasite infection process. Considering that Cav1 KO cells require EndoA2 expression to successfully repair most SLO lesions, as discussed above (Fig. 3B), these *T. cruzi* infection results provide independent evidence that cells lacking Cav1 become critically dependent on EndoA2 to survive PM injury – in this case inflicted by the parasites – as previously described (Fernandes et al., 2011).

The large size of *T. cruzi* trypomastigotes provided us with a good opportunity to examine EndoA2 localization during formation of the elongated, tight parasitophorous vacuoles that surround the parasites as they enter host cells. In order to detect early stages of the parasite-induced PM invaginations, we performed immunofluorescence assays of EndoA2 in WT and Cav1 KO MEFs incubated with *T. cruzi* for 15 min. Polyclonal anti-*T. cruzi* antibodies were used to stain extracellularly-exposed portions of the parasites prior to permeabilization of the cells and EndoA2 staining, allowing us to determine the stage of parasite invasion while assessing the recruitment of EndoA2.

**Fig. 6.**
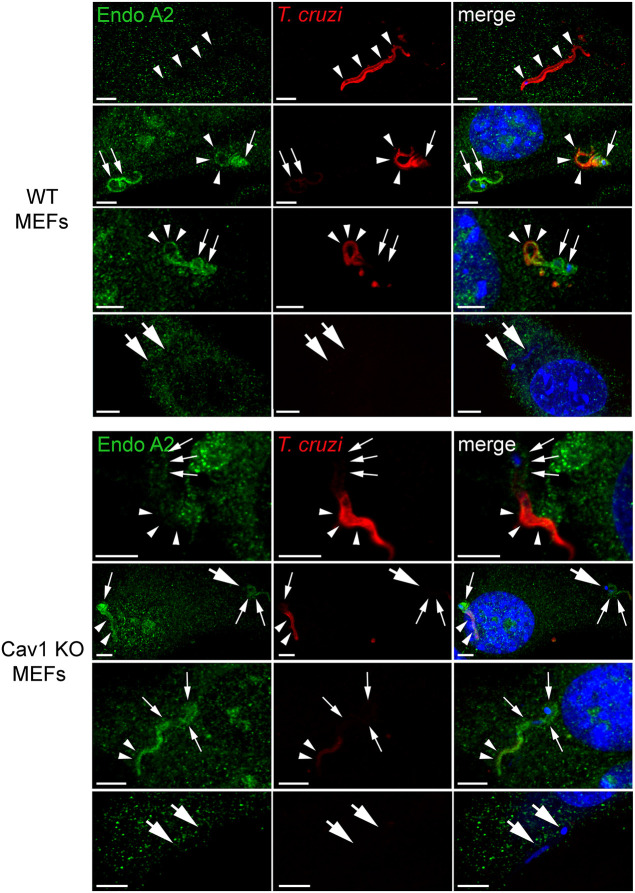
**EndoA2 is recruited to nascent parasite-containing vacuoles.** Confocal fluorescence microscopy images of EndoA2 staining (green) in WT and Cav1 KO MEFs infected with *T. cruzi* for 15 min. Extracellular or partially internalized *T. cruzi* parasites are stained with extracellularly-added anti-*T. cruzi* antibodies (red). Nuclei and parasite kinetoplast DNA are stained with DAPI (blue). Arrowheads: extracellular portions of *T. cruzi*. Small arrows: intracellular portions of *T. cruzi* within PM invaginations stained for EndoA2. Large arrows: intracellular *T. cruzi* showing fainter EndoA2 staining. Scale bars: 5 µm. Images are representative of three independent experiments.

In both WT and Cav1 KO MEFs, parasites that were fully stained with anti-*T. cruzi* antibodies and therefore considered extracellular showed no EndoA2 staining (Fig. 6, top panels). However, when partially internalized parasites were detected (Fig. 6, middle panels, arrowheads pointing to partial anti-*T. cruzi* extracellular staining), the internalized portion of the parasites was clearly surrounded by EndoA2 staining in both cell types (Fig. 6, middle panels, arrows pointing to EndoA2 staining). Interestingly, faint EndoA2 staining was visible on some parasite regions that were positive for anti-*T. cruzi* antibodies, suggesting that EndoA2 recruitment to the inner leaflet of the PM may be initiated prior to invasion. Fully internalized portions of the same parasites showed brighter staining with anti-EndoA2 antibodies, consistent with an accumulation of this cytosolic protein on the membrane of nascent parasitophorous vacuoles.

In several instances, fully internalized parasites showing no anti-*T. cruzi* staining were detected, and in this case continuous EndoA2 staining was observed along the tight intracellular compartments that are generated during cell invasion. However, a few fully internalized parasites negative for anti-*T. cruzi* staining did not react with EndoA2 antibodies (Fig. 6 WT and Cav1 KO MEFs, lower panels). This finding suggested that EndoA2 might only be transiently recruited to the *T. cruzi* parasitophorous vacuole, although this is difficult to assess for certain because the *T. cruzi* invasion process cannot be synchronized. These immunofluorescence results are consistent with a progressive recruitment of EndoA2 to the nascent *T. cruzi*-containing parasitophorous vacuoles, followed by EndoA2 loss after the host cell invasion process is completed. Thus, combined with the requirement for EndoA2 expression for cells to become susceptible to invasion by *T. cruzi*, our results identify EndoA2 as a component of the cellular machinery that is subverted by these parasites to invade host cells.

## DISCUSSION

Previous studies from our group have revealed a role for clathrin-independent endocytosis in the mechanism by which mammalian cells repair PM wounds. In NRK cells and muscle fibers, caveolar endocytosis is stimulated when cells are mechanically injured or permeabilized with the pore-forming toxin SLO, and Cav1 depletion inhibits caveolae internalization and PM repair. Direct visualization by electron microscopy of SLO within Cav1-containing intracellular vesicles suggested that caveolar endocytosis is directly involved in the removal and subsequent intracellular degradation of PM lesions (Corrotte et al., 2013; Corrotte et al., 2012). However, additional evidence implicating caveolae or other forms of clathrin-independent endocytosis in PM repair was needed, particularly in view of evidence that the ESCRT pathway can also promote cell resealing by removing small lesions from the PM (Jimenez et al., 2014).

B lymphocytes that lack Cav1 and caveolae can still reseal their PM after SLO permeabilization, concomitantly with upregulation of a tubular endocytic pathway (Miller et al., 2015). In this study we confirmed and expanded on those previous findings, providing mechanistic evidence for a role of tubular endocytosis in PM repair in cells lacking caveolae. Importantly, we found that expression of the endocytosis regulatory protein EndoA2 (Renard et al., 2015) was required for CTxB internalization and for the resealing of SLO-permeabilized MEFs, but only in Cav1-deficient fibroblasts lacking caveolae. In these cells, the most abundant CTxB carriers detected were tubular endosomes, and their scission from the PM required EndoA2, an effect that was not associated with increased EndoA2 expression levels (Fig. 3A). Notably, independent new evidence has uncovered a striking connection between B cell function and EndoA2-mediated PM repair. An unbiased CRISPR screen identified EndoA2 as an important regulator of B cell-mediated humoral responses (Malinova et al., 2020, preprint) and B cell PM wounding and repair emerged as an antigen-triggered process that facilitates antigen uptake and presentation to T cells (Maeda et al., 2020, preprint).

Overall, our findings are in line with the reported upregulation of clathrin-independent carriers known as CLIC/GEEC in Cav1 KO MEFs (Chaudhary et al., 2014), and suggest that when Cav1 is not present to shape the PM into structurally defined caveolae, formation of tubular endosomes capable of mobilizing similar lipid raft (cholesterol and sphingolipid-enriched) PM domains may be facilitated, with important functional consequences. Interestingly, our quantitative flow cytometry-based PM repair assay suggests that EndoA2-dependent tubular endocytosis is not as efficient as caveolar internalization in removing SLO pores from the PM, as indicated by the partial permeability to PI of Cav1 KO cell populations that recovered from SLO injury (Fig. 1).

In addition to strengthening the evidence for a role of endocytosis in PM repair, our findings uncovered a crosstalk between caveolar and EndoA2-dependent tubular endocytosis, consistent with previous suggestions (Chaudhary et al., 2014; Thottacherry et al., 2019). Our results also reinforce previous evidence that the intracellular parasite *T. cruzi* subverts the PM repair process to invade host cells (Fernandes et al., 2011). We found that Cav1 KO MEFs were slightly less susceptible to *T. cruzi* invasion, but when EndoA2 expression was silenced, parasite entry was markedly reduced in both WT and Cav1 KO MEFs. MEFs deficient in both Cav1 and EndoA2 could not survive longer periods of exposure to the parasites, in agreement with the strong PM repair defect observed in SLO-permeabilized cells under those conditions.

The PM repair and *T. cruzi* invasion processes have many properties in common. Both are initiated by Ca^2+^-triggered lysosomal exocytosis (Reddy et al., 2001; Tardieux et al., 1994; Tardieux et al., 1992), require PM cholesterol (Fernandes et al., 2007; Idone et al., 2008a; Tam et al., 2010) but not actin polymerization (Idone et al., 2008a; Tardieux et al., 1992), and are stimulated when cells are injured or treated with extracellular sphingomyelinase (Fernandes et al., 2011; Idone et al., 2008b). These properties, including stimulation by sphingomyelinase, are also associated with MEND, a massive endocytic process detected by electrophysiological approaches (Hilgemann et al., 2018). Interestingly, a recent study (Hubert et al., 2020) confirmed earlier findings that sphingomyelinase treatment stimulates caveolar endocytosis (Corrotte et al., 2013) and suggested that enrichment in glycosphingolipids and cholesterol resulting from sphingomyelin hydrolysis may promote caveolae fission from the PM through lipid phase separation mechanisms.

In order to remove PM lesions within the 12–15 s timeframe of PM repair (Idone et al., 2008b; Steinhardt et al., 1994), any endocytic uptake involved in this process would have to be fast. Notably, a fast form of tubular endocytosis (FEME) was previously identified and shown to be regulated by EndoA2 (Boucrot et al., 2015). The FEME pathway has several elements in common with the clathrin-independent tubular endocytic pathway that our study has linked to PM repair in Cav1 KO MEFs, and future studies should clarify its relationship with the CLIC/GEEC pathway (Howes et al., 2010a) found to be upregulated in Cav1 KO cells (Chaudhary et al., 2014). Proteins from the endophilin family have been shown to regulate the clathrin-independent uptake of various cargoes, including the IL2 receptor, β-adrenergic receptors and bacterial toxins (Boucrot et al., 2015; Renard et al., 2015; Chan Wah Hak et al., 2018).

Endophilins contain BAR domains capable of inducing membrane curvature, SH3 domains that bind cargo and multiple amphipathic helices proposed to support membrane scission (Boucrot et al., 2015; Gallop et al., 2006; Simunovic et al., 2017). A role for EndoA2 in fission of the tubular endosomes upregulated in Cav1 KO MEFs is supported by our quantitative ultrastructural analysis showing accumulation of CTxB-containing tubules connected to the PM and reduced CTxB internalization after EndoA2 depletion (Fig. 3). The frequent juxtaposition of CTxB and EndoA2 puncta that we observed by light microscopy in Cav1 KO MEFs (Fig. 4A) also agrees with a role for EndoA2 in endosome fission from the PM, consistent with previous studies proposing that the N-terminal α-helix of BAR proteins can induce strong membrane curvature, directly promoting membrane constriction and possibly fission (Boucrot et al., 2012; Farsad et al., 2001). Endophilins and other BAR family proteins bind dynamin through their SH3 domains, and this interaction was proposed to play a role in recruiting dynamin to endocytic vesicle necks to facilitate fission (Ferguson and De Camilli, 2012). However, a recent study showed that excess EndoA2 can intercalate between turns of the dynamin helix assembled on endosome necks, inhibiting membrane fission (Hohendahl et al., 2017). It is noteworthy, however, that previous studies found the removal of SLO toxin pores from the PM through caveolar endocytosis to be a dynamin-independent process (Corrotte et al., 2013; Idone et al., 2008b). Those findings are consistent with previous evidence indicating that clathrin-independent endocytosis is less reliant on dynamin-mediated fission (Howes et al., 2010b; Renard et al., 2015). Thus, additional studies are needed to clarify whether, in certain contexts, EndoA2 can act independently of dynamin to promote membrane fission.

In contrast to our evidence supporting a role of EndoA2 in the fission of tubular endosomes in Cav1-deficient cells, EndoA2 appeared to be gradually recruited to nascent parasitophorous vacuoles surrounding *T. cruzi* trypomastigotes during host cell entry. It is conceivable that in this scenario EndoA2 plays a predominant role in the extensive membrane deformation required for generating the large compartments surrounding these parasites during invasion. It is tempting to speculate that the ability of EndoA2 to polymerize into rigid scaffolds and block lipid diffusion, when under tension in membrane tubules (Simunovic et al., 2016), may participate in generation of the unusually tight and elongated parasitophorous vacuoles that surround intracellular *T. cruzi* parasites. Mounting evidence suggests that endophilins have broad functional roles in cells, acting as hubs for protein–protein interactions that coordinate several aspects of membrane remodeling (Kjaerulff et al., 2011; Saheki and De Camilli, 2012). The relatively slow and asynchronous *T. cruzi* host cell invasion process does not allow a precise determination of the invasion stage of individual parasites, but the fact that EndoA2 was not detected in some fully internalized parasites (Fig. 6) suggests that EndoA2 recruitment is transient, perhaps restricted to the early stages of invasion involving membrane deformation.

To our knowledge, this is the first time that a BAR domain-containing protein has been implicated in the extensive PM deformation that occurs during host cell invasion by *T. cruzi*. Our results identify EndoA2 as a novel molecular player in the PM remodeling process triggered by these parasites to enter host cells and in the compensatory tubular endocytic pathway that promotes PM repair in the absence of caveolae. In future studies it will be of great interest to investigate whether changes in lipid composition, such as those recently shown to stimulate caveolar endocytosis (Hubert et al., 2020), occur in caveolae and in tubular endosomes associated with PM repair and *T. cruzi* invasion.

## MATERIALS AND METHODS

### Cell culture

WT (ATCC, CRL 2752) and Cav1 KO (ATCC, CRL 2753) 3T3 MEFs were purchased from the American Type Culture Collection and cultured at 37°C in 5% CO_2_ in high glucose DMEM (Lonza, Alpharetta, GA, USA) containing 10% heat-inactivated FBS and penicillin/streptomycin (Invitrogen, Carlsbad, CA, USA). These cell lines were found to be free of mycoplasma contamination by ATCC and also through routine testing in the Andrews laboratory.

### Antibodies and reagents

The following antibodies and reagents were used to perform immunofluorescence, immunoblot and flow cytometry assays : rabbit anti-endophilin-A1 (36-3000; Life Technologies, Carlsbad, CA, USA); rabbit anti-endophilin-A3 (363200; Life Technologies); mouse anti-galectin-3 (sc-32790; Santa Cruz Biotechnology, Dallas, TX, USA); rabbit anti Alexa Fluor 488 (A11094; Invitrogen); mouse anti-endophilin-A2 (sc-365704; Santa Cruz Biotechnology); rabbit anti-clathrin heavy chain (ab21679; Abcam, Cambridge, UK), all at a 1:250 dilution; rabbit anti-*T cruz*i (Fernandes et al., 2011) at a 1:1000 dilution; rabbit anti Alexa Fluor 488 (A11094; Invitrogen) at 10 µg/ml dilution; mouse anti-endophilin-A2 (sc-365704; Santa Cruz Biotechnology) at 1:50 dilution; propidium iodide (PI) (P4170; Sigma-Aldrich, St. Louis, MO, USA); Alexa Fluor 488–cholera toxin B (C34775; Invitrogen); DAPI (D9542; Sigma-Aldrich) and Alexa Fluor-conjugated secondary antibodies (Invitrogen) at 1 µg/ml dilution.

### Flow cytometry PM repair assay

Histidine-tagged streptolysin-O (SLO) carrying a cysteine deletion that eliminates the need for thiol activation (provided by R. Tweten, University of Oklahoma, Norman, OK, USA), was expressed and purified as described in Idone et al. (2008b) and stored in 1 mg/ml aliquots at −80°C. For wounding with SLO, WT or Cav1 KO MEFs (treated or not with siRNA for 24–72 h) were trypsinized (0.25% trypsin at 37°C for 1–5 min) and split in two different suspensions in DMEM (with or without Ca^2+^) at the concentration of 1×10^5^ cells/ml. Aliquots of each cell suspension (0.1–0.2 ml) were incubated on ice with various concentrations of SLO for 5 min, transferred to a 37°C water bath for various time points (1–10 min) to induce pore formation followed by PM repair, and then transferred back to ice. To assess the degree of PM repair, the membrane impermeant dye PI (50 μg/ml) was added, and after 5 min at least 10,000 cells were analyzed by flow cytometry (FACS Canto II, Beckton Dickinson Biosciences, Sparks Glencoe, MD, USA).

### Imaging PM repair assay

WT and Cav1 KO MEFs (2.5×10^5^) were plated on 3.5 cm glass-bottom dishes (MatTek, Ashland, MA, USA) and 24 h later were treated with 100–400 ng/ml SLO for 5 min at 4°C followed by 5 min incubation in DMEM at 37°C, washed with phosphate-buffered saline (PBS) and stained for 5 min with 50 μg/ml PI, followed by washes with PBS and fixation in 4% paraformaldehyde (PFA) for 10 min. After staining with 10 μg/ml DAPI for 10 min, cells were washed and imaged immediately in a Zeiss LSM 980 Airyscan 2 laser scanning confocal microscope using a 20× 1.0 N.A. objective (Carl Zeiss microscopy, LLC, White Plains, NY, USA). Images were acquired randomly, and more than 200 DAPI-stained cells were visually scored as PI positive or not in each condition, in triplicate experiments.

### Transcriptional silencing

WT and Cav1 KO MEFs grown at ∼50% confluence were transfected with Mission siRNA transfection reagent (Sigma-Aldrich) and control, endophilin-A1, endophilin-A2, endophilin-A3, galectin-3 or clathrin heavy chain Silencer Select siRNA (Ambion, Life Technologies, Carlsbad, CA, USA), according to each manufacturer's instructions. After 24–72 h cells were used for various assays. Silencing of the target proteins was assessed by western blotting.

### Western blotting

Cell lysates were separated by SDS–PAGE and blotted onto nitrocellulose membranes using the Trans-Blot Transfer system (Bio-Rad Laboratories, Hercules, CA, USA). After incubation with primary antibodies followed by peroxidase-conjugated secondary antibodies, proteins were detected using Clarity Western ECL Substrate (Bio-Rad Laboratories) and a Fuji LAS-3000 Imaging System and Image Reader LAS-3000 software (Fuji, Edison, NJ, USA).

### Transmission electron microscopy

Sub-confluent cultures of WT and Cav1 KO MEFS on MatTek glass-bottom dishes (treated or not with EndoA2 siRNA for 48 h) were pre-incubated for 20 min in ice-cold DMEM with 5 µg/ml of CTxB–biotin (C34779; Invitrogen) coupled to streptavidin–gold (S9059; Sigma-Aldrich). Cell were then treated with 50 ng/ml SLO for 5 min at 4°C and further incubated for 1 min at 37°C in DMEM, before being embedded in resin *in situ* (no scraping and pelleting of fixed cells) and processed for TEM as previously described (Corrotte et al., 2013). Quantifications were performed by counting all vesicles containing CTxB–gold in 20 cell sections/sample, and scored as <100 nm vesicles, >100 nm vesicles, open or apparently closed tubular endosomes, or CCVs.

### Alexa Fluor 488–CTxB endocytosis

Subconfluent WT and Cav1 KO MEFs were treated or not with control or EndoA2 siRNA for 48 h, trypsinized, counted and diluted to 1×10^5^ cells/100 μl in DMEM. Cells were incubated with 5 µg/ml Alexa Fluor 488–CTxB at 4°C for 20 min, and then for 5 min at 4°C for with various concentrations of SLO. The cells were transferred (or not, for negative controls) to 37°C for 10 min, followed by transfer to 4°C and fixation in 4% PFA for 10 min. Flow cytometry was used to assess Alexa Fluor 488–CTxB fluorescence before and after quenching extracellular fluorescence by adding 10 μg/ml rabbit anti-Alexa Fluor 488 antibody (Life Technologies) and 1 mg/ml Trypan Blue (Sigma-Aldrich) at room temperature for 20 min. Data were analyzed using Flo-Jo software (Three Star Inc., Ashland, VA, USA).

### *Trypanosoma cruzi* infection

Trypomastigotes from the *T. cruzi* Y strain were obtained from the supernatant of infected monolayers of LLC-MK2 cells, as previously described (Tardieux et al., 1992). 1×10^5^ cells (WT or Cav1 KO MEFs) were plated on 3.5-cm dishes containing glass coverslips 48 h prior to experiments, and then treated or not with control or EndoA2 siRNA for 40 h prior to experiments. Coverslips with attached cells were incubated with trypomastigotes resuspended in 2 ml DME containing 2% FBS (multiplicity of infection 250–500) for the indicated periods of time at 37°C and washed five times with PBS to remove extracellular parasites. The coverslips were then fixed in 4% PFA for 15 min, washed three times with PBS and incubated for 15 min with 50 mM NH_4_Cl. Parasite and host cell DNA were stained for 10 min with 10 µM DAPI, and extracellular parasites were stained by incubation with rabbit anti-*T.cruzi* polyclonal antiserum (1:1000) for 30 min followed by anti-rabbit Alexa Fluor-conjugated secondary antibodies (Invitrogen). The number of intracellular parasites was determined by counting at least 200 cells per coverslip in triplicates, using a Nikon Eclipse E200 fluorescence microscope with a 100×1.3 N.A. oil immersion objective.

### Immunofluorescence and colocalization analysis

#### CTxB and EndoA2 localization

WT and Cav1 KO MEFs were cultured to 50% confluency on coverslips, pre-incubated with 5 µg/ml Alexa Fluor 488–CTxB for 20 min at 4°C and treated or not with 50 ng/ml of SLO for 5 min at 4°C. Cells were then incubated at 37°C in DMEM for 15 min before fixation in 4% PFA. Cells were then quenched with 50 mM NH_4_Cl for 15 min, blocked with 5% goat serum for 30 min and incubated with 10 μg/ml rabbit anti-Alexa Fluor 488 antibodies (Life Technologies) for 1 h to quench extracellular Alexa Fluor 488–CTxB fluorescence. Cells were then permeabilized with 0.2% saponin in PBS, blocked again with 5% goat serum in PBS containing 0.2% saponin, and incubated with mouse anti-endophilin-A2 (1:50) in PBS containing 0.2% saponin and 1% BSA overnight at 4°C, followed by an anti-mouse secondary antibody conjugated to Alexa Fluor 647 for 1 h and DNA staining with 10 μM DAPI. *Z* stack images (0.13 μm *Z* steps) were acquired using a confocal Leica SPX5 microscope with a 63×1.4 N.A. oil objective. Volocity software (PerkinElmer, Waltham, MA, USA) was used to apply a fine filter for noise reduction and to display each cell image in the XYZ mode. Overlapping signals between Alexa Fluor 647-labeled EndoA2 and Alexa Fluor 488–CTxB that could be visualized on all three axes were visually scored in at least 20 cells, in three independent experiments.

#### *Trypanosoma cruzi* and EndoA2 localization

WT and Cav1 KO MEFs were infected with *T. cruzi* trypomastigotes for 10–15 min, fixed with 4% PFA, quenched with 50 mM NH_4_Cl and blocked in PBS containing 5% goat serum, followed by incubation for 45 min with rabbit anti-*T. cruzi* serum (1:1000 in PBS containing 1% BSA) and Alexa Fluor 647-conjugated anti-rabbit IgG secondary antibodies for 45 min. Cells were then permeabilized with 0.2% saponin in PBS, and blocked again with PBS containing 5% goat serum and 0.2% saponin. Cells were then incubated with mouse anti-endophilin-A2 antibodies (1:50) in PBS containing 0.2% saponin and 1% BSA overnight at 4°C, followed by 1 h incubation with Alexa Fluor 488-conjugated anti-mouse secondary antibodies and DNA staining with 10 μM DAPI. *Z-*stack images (0.13 μm *Z* steps) were acquired using a confocal Leica SPX5 microscope with a 63×1.4 N.A. oil objective.

### Statistical analysis

Results obtained from two independent groups of cells (data acquisition and sample size details as provided above) were compared using unpaired, two-tailed Student's *t-*tests (Prism, GraphPad software). *P*-values <0.05 were considered significant. No samples were excluded from the analysis.

## Supplementary Material

Supplementary information

Reviewer comments
