# Supplementary material for: Endophilin-A2-dependent tubular endocytosis promotes plasma membrane repair and parasite invasion
Source: J Cell Sci. 2020 Dec 1;134(5):jcs249524. doi: 10.1242/jcs.249524 (PMC7725609; doi:10.1242/jcs.249524)
Supplement: Supplementary information [file joces-134-249524-s1.pdf]

Supplemental Figure 1

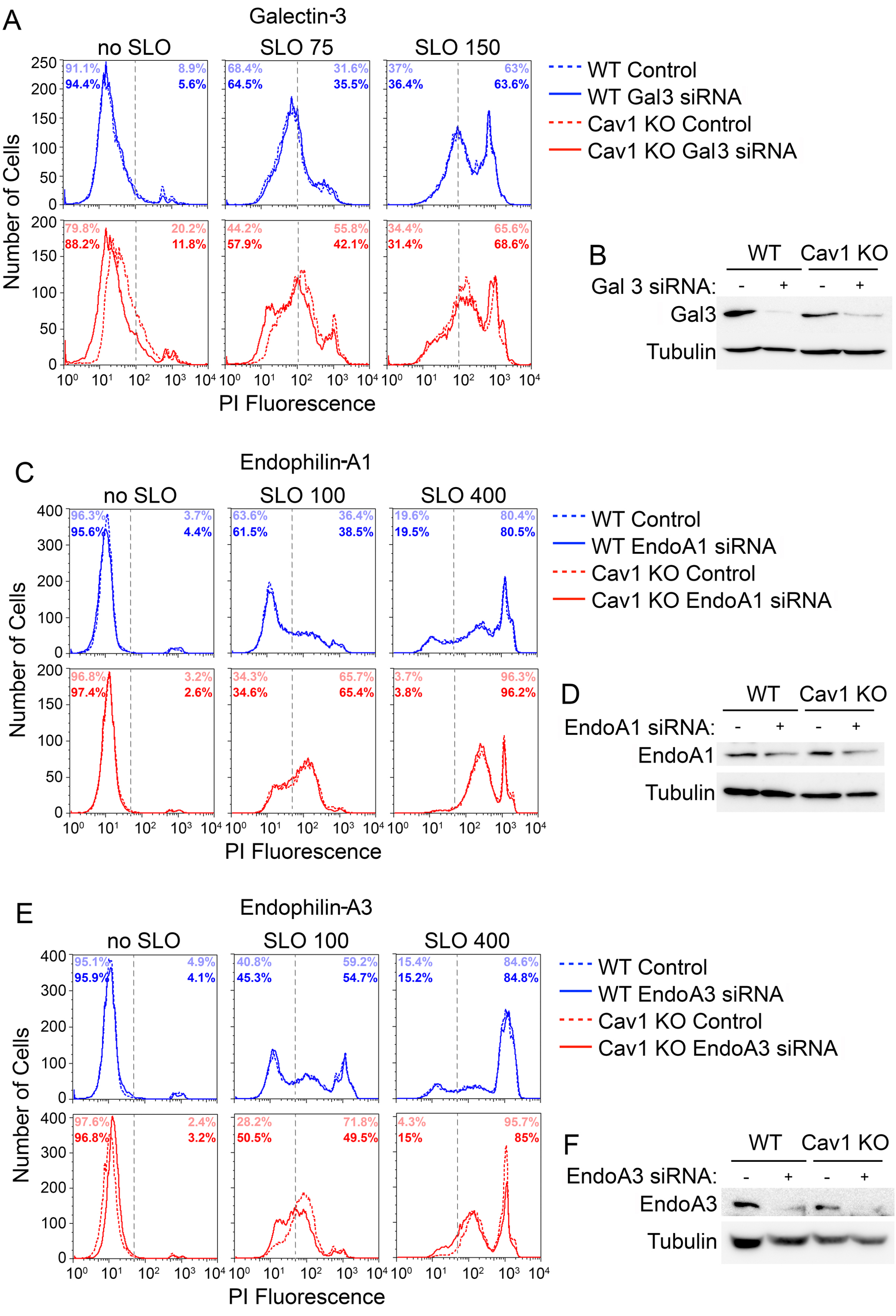

Journal of Cell Science • Supplementary information

Figure S1. Depletion of galectin-3, endophilin-A1 or endophilin A-3 does not inhibit PM repair in WT or Cav1 KO MEFs. A. Flow cytometry of WT (blue) and Cav1 KO MEFs (red) treated with control (dashed lines) or galectin-3 (full lines) siRNA for 48 h, exposed to increasing concentrations of SLO (75-150 ng/ml) +Ca^{2+} at 37°C for 2 min and stained with PI. The results are representative of 4 independent experiments. **B.** Western blot with antibodies against galectin-3 and tubulin (loading control) of WT and Cav1 KO MEF lysates treated with control or galectin-3 siRNA. **C.** Flow cytometry of WT (blue) and Cav1 KO MEFs (red) treated with control (dashed lines) or endophilin-A1 (full lines) siRNA for 48 h, exposed to increasing concentrations of SLO (100-400 ng/ml) +Ca^{2+} at 37°C for 2 min, and stained with PI. The results are representative of 4 independent experiments. **D.** Western blot with antibodies against endophilin-A1 and tubulin (loading control) of WT and Cav1 KO MEF lysates treated with control or endophilin-A1 siRNA. **E.** Flow cytometry of WT (blue) and Cav1 KO MEFs (red) treated with control (dashed lines) or endophilin-A3 (full lines) siRNA for 48 h, exposed to increasing concentrations of SLO (100-400 ng/ml) +Ca^{2+} at 37°C for 2 min, and stained with PI. The results are representative of 4 independent experiments. **F.** Western blot with antibodies against endophilin-A3 and tubulin (loading control) of WT and Cav1 KO MEF lysates treated with control or endophilin-A3 siRNA.

Journal of Cell Science • Supplementary information
