# Supplementary material for: Endophilin-A2-dependent tubular endocytosis promotes plasma membrane repair and parasite invasion
Source: J Cell Sci. 2020 Dec 1;134(5):jcs249524. doi: 10.1242/jcs.249524 (PMC7725609; doi:10.1242/jcs.249524)
Supplement: Reviewer comments [file joces-134-249524_review_history.pdf]

Endophilin-A2-dependent tubular endocytosis promotes plasma membrane repair and parasite invasion

Matthias Corrotte, Mark Cerasoli, Fernando Y. Maeda and Norma W. Andrews
DOI: 10.1242/jcs.249524

Editor: Derek Walsh

Review timeline

Original submission:	28 May 2020
Editorial decision:	10 July 2020
First revision received:	23 September 2020
Accepted:	12 October 2020

Original submission

First decision letter

MS ID#: JOCES/2020/249524

MS TITLE: Endophilin-A2-dependent tubular endocytosis promotes plasma membrane repair and parasite invasion

AUTHORS: Matthias Corrotte, Mark Cerasoli, and Norma W. Andrews
ARTICLE TYPE: Research Article

We have now reached a decision on the above manuscript.

To see the reviewers' reports and a copy of this decision letter, please go to: https://submit-jcs.biologists.org and click on the 'Manuscripts with Decisions' queue in the Author Area. (Corresponding author only has access to reviews.)

As you will see, the reviewers raise a number of substantial criticisms that prevent me from accepting the paper at this stage. They suggest, however, that a revised version might prove acceptable, if you can address their concerns. If you think that you can deal satisfactorily with the criticisms on revision, I would be pleased to see a revised manuscript. We would then return it to the reviewers.

We are aware that you may currently be unable to access the lab to undertake experimental revisions. If it would be helpful, we encourage you to contact us to discuss your revision in greater detail. Please send us a point-by-point response indicating where you are able to address concerns raised (either experimentally or by changes to the text) and where you will not be able to do so within the normal timeframe of a revision. We will then provide further guidance. Please also note that we are happy to extend revision timeframes as necessary.

Please ensure that you clearly highlight all changes made in the revised manuscript. Please avoid using 'Tracked changes' in Word files as these are lost in PDF conversion.

I should be grateful if you would also provide a point-by-point response detailing how you have dealt with the points raised by the reviewers in the 'Response to Reviewers' box. Please attend to all of the reviewers' comments. If you do not agree with any of their criticisms or suggestions please explain clearly why this is so.

© 2020. Published by The Company of Biologists under the terms of the Creative Commons Attribution License (https://creativecommons.org/licenses/by/4.0/).

Reviewer 1

Advance summary and potential significance to field

In this manuscript, M Corrotte et al., report on the contribution of the bar domain containing-endophilin (specifically the ubiquitous endophilin 2) to the uptake of membrane wounds caused in fibroblasts either by the cholesterol dependent pore-forming protein SLO or the invading protozoan Trypanosoma cruzi. The authors have previously shown that several cells including epithelial cells, fibroblasts and the injury prone muscle cells rely on the calveolae endocytosis to promote resealing of the plasma membrane at the site of SLO-triggered lesions and post membrane patch delivery through lysosome exocytosis. They now report that in fibroblasts derived from mouse homozygous for a disruption of the caveolinÂ-1 gene, and therefore lacking calveolae, an endophilin 2-dependent tubular endocytosis can also work as a suboptimal process to seal and repair the wound sites at the plasma membrane. By monitoring the cholera toxin internalization in SLO-exposed cells using quantitative EM analysis, the authors were able to establish the up regulation of the tubular endocytosis upon loss of caveolae whereas by silencing the endophilin-2 expression, they showed the contribution of endophilin-2-controlled tubular endocytosis in the repair of distinct membrane wounds. Two types of wounds at the fibroblast plasma membrane were analyzed, first the SLO toxin-driven pores (a classical model) or the membrane lesion caused at the site entry by the larger trypomastigote of the T. cruzi protozoan parasite.
The context and the rationale of the work are clearly presented including the elegant use of caveolin KO cells, although it might be slightly clarified for the working hypothesis (see below the specific comments). The experimental plan is well executed with appropriate approaches including quantitative FACS analysis and qEM imaging as well confocal imaging. The data convincingly document that silencing endophilin2 in caveolae-deficient MEF cells significantly increases the membrane wound extent (i.e. decreases the resealing capacity as readily assessed by influx of propidium iodide in a statistically relevant sample of cells). If the use of the SLO system is well introduced, the added value of the T. cruzi invading trypomastigote as a living probe for membrane repair could be more emphasized. Another issue that could be discussed in more depth concerns the still complex conclusions about the endophilin2 involvement in the two distinct membrane-resealing processes analyzed here.
Overall considering that the coupling between membrane repair and the exocytosis and endocytosis processes is widely investigated and thus of clear interest for the broad readership of Journal of cell Science, this well written manuscript, with no overstatements brings an additional and original piece of evidence of the machinery characterizing the Fast Endophilin Mediated Endocytosis (FEME) being mobilized during membrane repair upon distinct membrane wounding events. In addition, the endophilin2-tubular endocytosis as an additional mechanism hijacked by T. cruzi trypomastigote to promote efficient entry while critically ensuring host cell survival should interest the cell biologists -to which I belong- that acknowledge protozoan intracellular microbes as great living probes to unravel fundamental processes of the metazoan cells.
I would therefore recommend the manuscript to be accepted for publication in JCS once the minor following issues will be addressed.

Comments for the author

Minor comments:
- Page 4
- « An investigation of the mechanism by which mammalian cells reseal after attack by pore-forming toxins revealed that streptolysin O (SLO) pores …”
The sentence could be better phrased since the pore-forming toxins first mentioned are plural and raise a general idea and then the sentence continues with the specific SLO.
-« In this study we investigated this hypothesis by examining mouse embryonic fibroblasts… I think this sentence about the working hypothesis is slightly confusing. The study does not directly address the “possibility that when proteins necessary for the assembly of caveolae are absent, lipid raft PM microdomains may be mobilized for internalization in the form of larger, tubule-shaped endosomes”. Rather the study is centered around a late step of the PM repair process (i.e. uptake of a wounded membrane pieces) and the authors start by showing that the lack of caveolae translates into a delayed:impaired membrane sealing event. Then, the study shows the increased

© 2020. Published by The Company of Biologists under the terms of the Creative Commons Attribution License (https://creativecommons.org/licenses/by/4.0/).

tubular endocytosis in the caveolae-deficient cells that correlates with the direct endophilin functional contribution to the PM repair processes they investigate.
Page 6
– “to examine recently formed intracellular compartments containing CTxB, a well-established cargo of clathrin-independent endocytosis” It seems to me that in fact the internalization of CT xB was reported to be mediated by all three Clathrin- Caveolin- and Arf6-dependent Pathways. There is no doubt it is good tracer for assessing calveolae and the « compensatory » tubular endocytosis but it might useful to clarify the sentence (it is actually implcit in the Figure 2B with a minor contribution of CCV to the CTxB gold distribution and also mentioned in the text with another assay targeting the CME, data not shown).
Page 7
– « These results suggest that Caveolin 1 deficiency in MEFs is associated with upregulation of a population of clathrin-independent tubular endosomes perhaps as a compensatory mechanism for the absence of caveolae.” May be it would be nice to refer here to the similarity with the lymphocyte B case. I have tried to search if other leucocytes or cells (beside the case of the red blood cells) constitutively lack the caveolae pathway and display instead a prominent tubular-endocytosis pathway but did not find any. I was also wondering what is known about mechanisms regulating the tubular endocytosis in cells (not only through the impairment of caveolae endocytic route).
This will lead to my first questioning about the physiological importance of the tubular endocytosis-dependent repair system that might be addressed in the discussion. It does not weaken the interest of the present dissection of an “alternative/compensatory” mechanism of membrane wound uptake and healthy membrane resealing. My interpretation based on the increased (significant?, Figure 3D) of CTxB tubules in the EndoA2 WT cells is that the EndoA2 silencing might freeze the dynamics of tubules thus reflecting a Endo2 contribution in promoting the late fission event of the otherwise fast FEME occurring in caveolae-positive cells but this is not so clear since both apparent close and open tubes are possibly increased in absence of Endo2A in these WT cells (I am not sure the differences are statistically valid but are not shown here). Clearly, this endophilin-mediated fission conclusion fits well with the results for the Cav1 null cells. Moreover, the view of a contribution of EndoA2 to membrane fission, which is one result stressed by the authors, is not sufficient to explain the observation of the Endo2-positive nascent vacuole surrounding the invading trypomastigote (even in area where the trypo body is not yet internalized). While this is addressed later in the discussion it might be good to expand on what is known of the function of endophilinA2, and for instances on the possible functional interplay with the dynamin fission activity (there is a interesting recent study that might be worth to cite: Hoehendahl et al., Elife 2017; 6:e26856).
I would propose to introduce these concerns in page 12 results because it would nicely support the justification of the T. cruzi/endoA2 visualization.“The large size of T. cruzi trypomastigotes provided us with a good opportunity to examine EndoA2 localization during formation of the elongated, large parasitophorous vacuoles that surround the parasites as they enter host cells. In order to detect early stages of the parasite-induced PM invaginations, we performed immunofluorescence assays of EndoA2 in WT and Cav1 KO MEFs incubated with T. cruzi for 15 min”.
Page 8
- “On the other hand, RNAi-mediated knock-down of endophilin-A2 (EndoA2) significantly increased the number of PI-positive Cav1 KO cells when compared to controls, revealing a PM repair defect”. The phrasing could be improved because there is already a PM repair defect. I would suggest that what the assays show is an “accentuation” of the repair defect.

Figure 1 :
A and B- would be it be possible to indicate the range of the total number of cells analyzed by FACS on the representative assays shown. Same for figure 3A C- please add an intensity scale since it is not only the number of PI cells that is interesting but the amount of fluorescence (that can be detected in both the nucleus and the cytoplasm) that nicely accounts for the extent of permeabilization (i.e. repair failures). Please mentioned for exemple a ratio of PI positive detected cells (for total cells around 100, which would be about 3 fields of view)
Figure 3 May be consider to change the B (Western result) before the A. It would be also interesting to mention that in spite of the increasing tubular endosomes in the Cav1 KO cells, there is no overexpression of endophilin2

© 2020. Published by The Company of Biologists under the terms of the Creative Commons Attribution License (https://creativecommons.org/licenses/by/4.0/).

(see my previous comment on the work of Hoehendahl et al., Elife 2017 where overexpression of endoA2 is shown impairing the endocytic uptake). In the current Figure 3, I noticed the significant effect of Endo2 silencing on the repair efficacy in WT exposed to 75 ng/ml of SLO. In the text, the authors were careful and correct by specifying that « no consistent inhibition of PM repair was observed in WT MEFs after reducing EndoA2 expression levels"
but does this increase hold for the 75 ng/ml dose for the 4 experiments mentioned in the legend?
Figure 4
If possible it would be nice to provide quantitative analysis of the amount of endoA2 and CTxB IF signal juxtaposition to appreciate the increase of tubular endocytosis in the Cav1 lacking cells.

Reviewer 2

Advance summary and potential significance to field

In this article, Corotte et al. examine the role of endophilin-A2 in plasma membrane repair. The authors show that caveolin-1-deficient fibroblasts have a reduced capacity to reseal after challenge with pore-forming toxins. Silencing endophilin-A2 expression inhibited fission of endocytic tubules and further reduced plasma membrane repair in cells lacking caveolin-1. Immuno-staining experiments revealed Endophilin-A2 in association with cholera toxin B-containing endosomes. Endophilin-A2 was also recruited to the vacuoles formed upon Trypanosoma cruzi invasion. Endophilin-A2 depletion decreased T. cruzi invasion and silencing of Endophilin-A2 in fibroblasts lacking caveolin-1 affected the ability of infected cells to survive infection. The authors conclude that in absence of caveolin-1, endophilin-A2 supports clathrin-independent endocytosis and plasma membrane repair, as well as T. cruzi invasion.

Comments for the author

This is an interesting study and the results are clearly presented. The only criticism would be the lack of quantification and/or statistical analysis in some of the reported experiments. These include:

Figure 1C: although the difference between wild type and cav1 KO cells is obvious by visual inspection, it would be nice to have some sort of quantification for this assay.

Figure 3 A and 3E: there is no statistical analysis. How significant are the claimed differences?

Figure 4: there is no quantification in support of "Cav1 KO MEFs showed higher levels of these partially colocalized CTxB-EndoA2 structures at both time points".

Figure 6: it would be nice to provide some sort of numeration for the scored phenotypes. For instance, among all partially internalized parasites, how many displayed an internalized portion positive endoA2 staining? Wordings such as "in several instances"..."a few internalized parasites"... suggest the feasibility of quantitative evaluation.

Reviewer 3

Advance summary and potential significance to field

I am sorry for my late response. I is unfortunate that the authors mix two models in this study. Each of the models needs to be handled with care and the lack of this is a major drawback of the paper. Also, I am not sure whether all data is truly representative (as they repeated experiments only twice in some cases).

Comments for the author

This manuscript is about wound repair of plasma membranes using different models for membrane damage/perturbation. The authors start with caveolin-1 knockout fibroblasts and their capacity to repair the damage of pore forming toxins.

© 2020. Published by The Company of Biologists under the terms of the Creative Commons Attribution License (https://creativecommons.org/licenses/by/4.0/).

Through this they make a link with endophilin-A2 through knockdown studies in cells that have already been depleted for caveolin-1. The endophilin implication was further studied in a Trypanosoma cruzi model system that is also exploiting membrane repair pathways.

Together, the authors make links between caveolin-1 and endophilin-A2 regulating clathrin-independent endocytosis and membrane repair. The presented data is not always clear, especially with regards to the electron microscopy. Also, the authors switch between different models for membrane damage and repair and different cell types. This makes the interpretation of the data difficult and it is not easy what is the message the authors wish to convey. I would have preferred to have a careful characterization of either model system, but not a mixture between the two. Also the presented data is of poor quality and it is not clear whether the shown images are actually representative.

The main problem is that this study uses different model systems, such as pore forming toxins and the parasite Trypanosoma cruzi to draw general conclusions on two trafficking pathways and their role in membrane repair.

It was also difficult for me to understand how the quantifications were done in figure 2 and figure 3 (it's not clear in the methods). I actually think that the proposed quantification is impossible as it depends on the sample preparation and there may be quite some variations.

Figure 4 is based on two experiments. This does not allow to draw an conclusions. Also, it is not described how the data was chosen and wether it is representative.

Knockouts should be complemented. No controls have been performed in this direction. The double perturbations performed by the authors need to be analyzed with care.

Only at the end of the discussion, it became clear what the authors wanted to convey. It would have been appreciated to make the main points on the BAR domain proteins stronger throughput, so that the reader can also follow better the choice of the models.

First revision

Author response to reviewers' comments

Point-by-point Response to Reviewers:

Reviewer 1 Advance Summary and Potential Significance to Field:
In this manuscript, M Corrotte et al., report on the contribution of the bar domain containing-endophilin (specifically the ubiquitous endophilin 2) to the uptake of membrane wounds caused in fibroblasts either by the cholesterol dependent pore-forming protein SLO or the invading protozoan Trypanosoma cruzi. The authors have previously shown that several cells including epithelial cells, fibroblasts and the injury prone muscle cells rely on the calveolae endocytosis to promote resealing of the plasma membrane at the site of SLO-triggered lesions and post membrane patch delivery through lysosome exocytosis. They now report that in fibroblasts derived from mouse homozygous for a disruption of the caveolin1 gene, and therefore lacking calveolae, an endophilin 2-dependent tubular endocytosis can also work as a suboptimal process to seal and repair the wound sites at the plasma membrane. By monitoring the cholera toxin internalization in SLO- exposed cells using quantitative EM analysis, the authors were able to establish the up regulation of the tubular endocytosis upon loss of caveolae whereas by silencing the endophilin-2 expression, they showed the contribution of endophilin-2-controlled tubular endocytosis in the repair of distinct membrane wounds. Two types of wounds at the fibroblast plasma membrane were analyzed, first the SLO toxin-driven pores (a classical model) or the membrane lesion caused at the site entry by the larger trypomastigote of the T. cruzi protozoan parasite.

The context and the rationale of the work are clearly presented including the elegant use of

© 2020. Published by The Company of Biologists under the terms of the Creative Commons Attribution License (https://creativecommons.org/licenses/by/4.0/).

caveolin KO cells, although it might be slightly clarified for the working hypothesis (see below the specific comments). The experimental plan is well executed with appropriate approaches including quantitative FACS analysis and qEM imaging as well confocal imaging. The data convincingly document that silencing endophilin2 in caveolae-deficient MEF cells significantly increases the membrane wound extent (i.e. decreases the resealing capacity as readily assessed by influx of propidium iodide in a statistically relevant sample of cells). If the use of the SLO system is well introduced, the added value of the T. cruzi invading trypomastigote as a living probe for membrane repair could be more emphasized.

RESPONSE: We agree, and have added a section in the introduction to better explain the parallels between *T. cruzi* invasion and PM repair, citing our prior studies in this area. We thank the reviewer for this suggestion, which has greatly improved the flow of our manuscript.

Another issue that could be discussed in more depth concerns the still complex conclusions about the endophilin2 involvement in the two distinct membrane-resealing processes analyzed here.

RESPONSE: Following up on this suggestion, we have extended our discussion of these issues in the Discussion section of the manuscript.

Overall considering that the coupling between membrane repair and the exocytosis and endocytosis processes is widely investigated and thus of clear interest for the broad readership of Journal of cell Science, this well written manuscript, with no overstatements brings an additional and original piece of evidence of the machinery characterizing the Fast Endophilin Mediated Endocytosis (FEME) being mobilized during membrane repair upon distinct membrane wounding events. In addition, the endophilin2- tubular endocytosis as an additional mechanism hijacked by T. cruzi trypomastigote to promote efficient entry while critically ensuring host cell survival should interest the cell biologists -to which I belong- that acknowledge protozoan intracellular microbes as great living probes to unravel fundamental processes of the metazoan cells.
I would therefore recommend the manuscript to be accepted for publication in JCS once the minor following issues will be addressed.

RESPONSE: We greatly appreciate the positive comments, and in the revised manuscript have addressed all suggestions, including the addition of more experimental data in Figs. 1 and 4.

Reviewer 1 Comments for the Author:
Minor comments:
- Page 4
- « An investigation of the mechanism by which mammalian cells reseal after attack by pore-forming toxins revealed that streptolysin O (SLO) pores ...”
The sentence could be better phrased since the pore-forming toxins first mentioned are plural and raise a general idea and then the sentence continues with the specific SLO.

RESPONSE: That sentence was rephrased, and now reads: "*An investigation of the mechanism by which mammalian cells reseal after attack by the pore-forming toxin streptolysin O (SLO) revealed that toxin pores can be internalized within <100 nm caveolar vesicles (Corrotte et al., 2013) and trafficked to lysosomes for degradation (Corrotte et al., 2012).*"

-« In this study we investigated this hypothesis by examining mouse embryonic fibroblasts. I think this sentence about the working hypothesis is slightly confusing. The study does not directly address the "possibility that when proteins necessary for the assembly of caveolae are absent, lipid raft PM microdomains may be mobilized for internalization in the form of larger, tubule-shaped endosomes”. Rather the study is centered around a late step of the PM repair process (i.e. uptake of a wounded membrane pieces) and the authors start by showing that the lack of caveolae translates into a delayed:impaired membrane sealing event. Then, the study shows the increased tubular endocytosis in the caveolae-deficient cells that correlates with the direct endophilin functional contribution to the PM repair processes they investigate.

RESPONSE: We thank the reviewer for this relevant comment. We have rephrased that statement which now reads: *“In this study we have extended our investigation of PM repair in caveolae-*

© 2020. Published by The Company of Biologists under the terms of the Creative Commons Attribution License (https://creativecommons.org/licenses/by/4.0/).

deficient cells by examining "

Page 6

- "to examine recently formed intracellular compartments containing CTxB, a well-established cargo of clathrin-independent endocytosis" It seems to me that in fact the internalization of CT xB was reported to be mediated by all three Clathrin- Caveolin- and Arf6-dependent Pathways. There is no doubt it is good tracer for assessing calveolae and the « compensatory » tubular endocytosis but it might useful to clarify the sentence (it is actually implcit in the Figure 2B with a minor contribution of CCV to the CTxB gold distribution and also mentioned in the text with another assay targeting the CME, data not shown).

RESPONSE: That statement has been clarified, and now reads: "*CTxB is a well- established cargo of clathrin-independent endocytosis, but can also enter cells through additional endocytic pathways (Stoeber 2012).*"

Page 7

- « These results suggest that Caveolin 1 deficiency in MEFs is associated with upregulation of a population of clathrin-independent tubular endosomes, perhaps as a compensatory mechanism for the absence of caveolae." May be it would be nice to refer here to the similarity with the lymphocyte B case. I have tried to search if other leucocytes or cells (beside the case of the red blood cells) constitutively lack the caveolae pathway and display instead a prominent tubular-endocytosis pathway but did not find any. I was also wondering what is known about mechanisms regulating the tubular endocytosis in cells (not only through the impairment of caveolae endocytic route).

RESPONSE: We thank the reviewer for this suggestion, and have added a reference to the findings in B cells to this sentence, which now reads: "*These results suggest that Cav1 deficiency in MEFs is associated with upregulation of a population of clathrin- independent tubular endosomes, perhaps as a compensatory mechanism for the absence of caveolae as previously observed in B lymphocytes (Miller et al., 2015).*" We have also added citations to two recent BioRxiv preprints that we are very excited about - an independent link between endophilin A2 and plasma membrane repair that emerged from an unbiased CRISPR screen for endocytic molecules that affect B cell function (Maeda et al. 2020, and Malinova et al. 2020).

This will lead to my first questioning about the physiological importance of the tubular endocytosis-dependent repair system that might be addressed in the discussion. It does not weaken the interest of the present dissection of an "alternative/compensatory" mechanism of membrane wound uptake and healthy membrane resealing. My interpretation based on the increased (significant?, Figure 3D) of CTxB tubules in the EndoA2 WT cells is that the EndoA2 silencing might freeze the dynamics of tubules thus reflecting a Endo2 contribution in promoting the late fission event of the otherwise fast FEME occurring in caveolae-positive cells but this is not so clear since both apparent close and open tubes are possibly increased in absence of Endo2A in these WT cells (I am not sure the differences are statistically valid but are not shown here). Clearly, this endophilin-mediated fission conclusion fits well with the results for the Cav1 null cells. Moreover, the view of a contribution of EndoA2 to membrane fission, which is one result stressed by the authors, is not sufficient to explain the observation of the Endo2- positive nascent vacuole surrounding the invading trypomastigote (even in area where the trypo body is not yet internalized). While this is addressed later in the discussion it might be good to expand on what is known of the function of endophilinA2, and for instances on the possible functional interplay with the dynamin fission activity (there is a interesting recent study that might be worth to cite: Hoehendahl et al., Elife 2017; 6:e26856).
I would propose to introduce these concerns in page 12 results because it would nicely support the justification of the T. cruzi/endoA2 visualization."The large size of T. cruzi trypomastigotes provided us with a good opportunity to examine EndoA2 localization during formation of the elongated, large parasitophorous vacuoles that surround the parasites as they enter host cells. In order to detect early stages of the parasite- induced PM invaginations, we performed immunofluorescence assays of EndoA2 in WT and Cav1 KO MEFs incubated with T. cruzi for 15 min".

RESPONSE: These are indeed valid points and we have now addressed them in our expanded discussion. Answering the reviewer's specific comments about Fig. 3D, only the comparisons marked

© 2020. Published by The Company of Biologists under the terms of the Creative Commons Attribution License (https://creativecommons.org/licenses/by/4.0/).

with asterisks and a P value were found to be statistically significant.

Page 8
- “On the other hand, RNAi-mediated knock-down of endophilin-A2 (EndoA2) significantly increased the number of PI-positive Cav1 KO cells when compared to controls, revealing a PM repair defect”. The phrasing could be improved because there is already a PM repair defect. I would suggest that what the assays show is an “accentuation” of the repair defect.

RESPONSE: We fully agree, thanks for the suggestion! That sentence now reads: “*On the other hand, RNAi-mediated knock-down of endophilin-A2 (EndoA2) significantly increased the number of PI-positive Cav1 KO cells when compared to controls, accentuating the PM repair defect observed in these cells.* ”

Figure 1 :
A and B- would be it be possible to indicate the range of the total number of cells analyzed by FACS on the representative assays shown. Same for figure 3A

RESPONSE: This is indicated in Materials and Methods - more than 10,000 cells per condition were analyzed in all flow cytometry assays.

C- please add an intensity scale since it is not only the number of PI cells that is interesting but the amount of fluorescence (that can be detected in both the nucleus and the cytoplasm) that nicely accounts for the extent of permeabilization (i.e. repair failures). Please mentioned for exemple a ratio of PI positive detected cells (for total cells around 100, which would be about 3 fields of view)

RESPONSE: We have chosen to quantify the number of PI positive cells in this experiment, since this was also requested by another reviewer - the results are now shown in the **new Fig. 1C and 1D**. We agree that the total amount of PI entering cells is an interesting parameter, but the use of an intensity scale in this case is problematic because the PI fluorescence intensity increases markedly after intercalation into DNA.

Figure 3
May be consider to change the B (Western result) before the A. It would be also interesting to mention that in spite of the increasing tubular endosomes in the Cav1 KO cells, there is no overexpression of endophilin2 (see my previous comment on the work of Hoehendahl et al., Elife 2017 where overexpression of endoA2 is shown impairing the endocytic uptake).

RESPONSE: Thanks for the suggestions. We have changed the order of Fig. A and B as suggested, and now mention that result in our revised Discussion.

In the current Figure 3, I noticed the significant effect of Endo2 silencing on the repair efficacy in WT exposed to 75 ng/ml of SLO. In the text, the authors were careful and correct by specifying that « no consistent inhibition of PM repair was observed in WT MEFs after reducing EndoA2 expression levels” but does this increase hold for the 75 ng/ml dose for the 4 experiments mentioned in the legend?

RESPONSE: No, this slight reduction in PI positive cells shown in Fig. 3A with 75 ng/ml SLO was not consistently observed in all 4 experiments, and that is why we did not specifically emphasize it.

Figure 4
If possible it would be nice to provide quantitative analysis of the amount of endoA2 and CTxB IF signal juxtaposition to appreciate the increase of tubular endocytosis in the Cav1 lacking cells.

RESPONSE: We agree, and now show in the **new Fig. 4B** the results of quantification of the juxtaposition between EndoA2 and CTxB puncta using stringent criteria (overlapping regions on all 3 XYZ axis of the confocal Z stack).

Reviewer 2 Advance Summary and Potential Significance to Field:
In this article, Corotte et al. examine the role of endophilin-A2 in plasma membrane repair. The

© 2020. Published by The Company of Biologists under the terms of the Creative Commons Attribution License (https://creativecommons.org/licenses/by/4.0/).

authors show that caveolin-1-deficient fibroblasts have a reduced capacity to reseal after challenge with pore-forming toxins. Silencing endophilin-A2 expression inhibited fission of endocytic tubules and further reduced plasma membrane repair in cells lacking caveolin-1. Immuno-staining experiments revealed Endophilin-A2 in association with cholera toxin B-containing endosomes. Endophilin-A2 was also recruited to the vacuoles formed upon Trypanosoma cruzi invasion. Endophilin-A2 depletion decreased T. cruzi invasion, and silencing of Endophilin-A2 in fibroblasts lacking caveolin-1 affected the ability of infected cells to survive infection. The authors conclude that in absence of caveolin-1, endophilin-A2 supports clathrin-independent endocytosis and plasma membrane repair, as well as T. cruzi invasion.

Reviewer 2 Comments for the Author:
This is an interesting study and the results are clearly presented. The only criticism would be the lack of quantification and/or statistical analysis in some of the reported experiments. These include:

Figure 1C: although the difference between wild type and cavl KO cells is obvious by visual inspection, it would be nice to have some sort of quantification for this assay.

RESPONSE: We have added this quantification, done in more than 200 cells in triplicate assays. The results are now shown in the **new Fig. 1C and 1D**.

Figure 3 A and 3E: there is no statistical analysis. How significant are the claimed differences?

RESPONSE: As we stated above and in the manuscript, the FACS profiles shown in Fig. 3A and 3E are representative of multiple experiments, and in each experiment more than 10,000 cells were analyzed for each condition. Below we show the requested statistical analysis, which confirms the increased PI permeability of Cav1 KO cells treated with EndoA2 siRNA.

Comparison between the average number of PI-positive cells in six independent experiments:
The P values comparing WT cells treated with control and EndoA2 siRNA were all above 0.195.

NOTE: We have removed unpublished data that had been provided for the referees in confidence.

Comparison between the average fluorescence intensity of internalized CTxB in three independent experiments:
The P values comparing WT cells treated with control and EndoA2 siRNA were all above 0.116.

NOTE: We have removed unpublished data that had been provided for the referees in confidence.

Figure 4: there is no quantification in support of "Cav1 KO MEFs showed higher levels of these partially colocalized CTxB-EndoA2 structures at both time points".

RESPONSE: We have added this quantification in the **new Fig. 4B**, done using stringent criteria for juxtaposition (overlapping regions on all 3 XYZ axis of the confocal Z stack).

Figure 6: it would be nice to provide some sort of numeration for the scored phenotypes. For instance, among all partially internalized parasites, how many displayed an internalized portion positive endoA2 staining? Wordings such as "in several instances"..."a few internalized parasites"... suggest the feasibility of quantitative evaluation.

RESPONSE: Unfortunately the *T. cruzi* invasion process is slow and asynchronous. It takes several minutes for these large and highly motile parasites to attach and start moving into host cells. Unfortunately, this process cannot be synchronized because the time required for individual parasites to attach to cells is highly variable, and not all attachment events are productive (leading to cell invasion). For this reason, it is not possible to reliably perform quantifications of each invasion stage. We have thus restricted our analysis to morphological assessments based on our extensive prior experience studying these parasites.

Reviewer 3 Advance Summary and Potential Significance to Field:
I am sorry for my late response. I is unfortunate that the authors mix two models in this study. Each

© 2020. Published by The Company of Biologists under the terms of the Creative Commons Attribution License (https://creativecommons.org/licenses/by/4.0/).

of the models needs to be handled with care and the lack of this is a major drawback of the paper. Also, I am not sure whether all data is truly representative (as they repeated experiments only twice in some cases).

Reviewer 3 Comments for the Author:
This manuscript is about wound repair of plasma membranes using different models for membrane damage/perturbation. The authors start with caveolin-1 knockout fibroblasts and their capacity to repair the damage of pore forming toxins. Through this they make a link with endophilin-A2 through knockdown studies in cells that have already been depleted for caveolin-1. The endophilin implication was further studied in a Trypanosoma cruzi model system that is also exploiting membrane repair pathways.

Together, the authors make links between caveolin-1 and endophilin-A2 regulating clathrin-independent endocytosis and membrane repair. The presented data is not always clear, especially with regards to the electron microscopy. Also, the authors switch between different models for membrane damage and repair and different cell types. This makes the interpretation of the data difficult and it is not easy what is the message the authors wish to convey. I would have preferred to have a careful characterization of either model system, but not a mixture between the two.

RESPONSE: We hope that our extended introduction and discussion have explained better the message conveyed in this manuscript, which is a logical follow-up of extensive studies made in our laboratory on both *T. cruzi* invasion and the mechanism of plasma membrane repair.

Also the presented data is of poor quality and it is not clear whether the shown images are actually representative.

RESPONSE: All images shown are highly representative of our findings, as stated in the manuscript. In our **new Fig. 1B**, we replaced the original images by new ones that are representative of 3 new experiments, and quantification of these new experiments revealed highly significant differences.

The main problem is that this study uses different model systems, such as pore forming toxins and the parasite Trypanosoma cruzi to draw general conclusions on two trafficking pathways and their role in membrane repair.

RESPONSE: We hope that our revised text is now more clear on the previously established relationship between these two processes.

It was also difficult for me to understand how the quantifications were done in figure 2 and figure 3 (it's not clear in the methods). I actually think that the proposed quantification is impossible as it depends on the sample preparation and there may be quite some variations.

RESPONSE: The quantifications were done following established methods in quantitative electron microscopy. We have now added more details to the Materials and Methods section to make it clearer that our cells were embedded for TEM while still attached to the coverslips, providing a highly reproducible orientation of the cells for sectioning. We also kept a reference that we had originally included to our prior publication in eLife (2013) that made extensive use of this methodology.
Figure 4 is based on two experiments. This does not allow to draw an conclusions. Also, it is not described how the data was chosen and whether it is representative.

RESPONSE: In the **new Fig. 4B** we now provide quantification and statistical analysis between the results of 3 independent experiments. The results show a statistically significant increase in juxtaposition events between EndoA2 and CTxB puncta.

Knockouts should be complemented. No controls have been performed in this direction. The double perturbations performed by the authors need to be analyzed with care.

RESPONSE: We agree that complementation of Cav1 KO cells would in principle have been informative. Unfortunately, complementation of caveolin-1 is a very challenging process, because even small overexpressions have severe impact in cellular function (Hayer, A. et al. J. Cell Biology

© 2020. Published by The Company of Biologists under the terms of the Creative Commons Attribution License (https://creativecommons.org/licenses/by/4.0/).

191:615-629, 2010). The Helenius group showed clearly in this study that increased levels of Cav1 saturates the intracellular trafficking of this protein, leading to redirection of Cav1 not associated with caveolae to lysosomes for degradation. This finding led them to revise their original proposal of a novel organelle caused caveosome - it turned out that what they had observed was an unusual accumulation of ectopically expressed Cav1 in late endosomes/lysosomes.

Only at the end of the discussion, it became clear what the authors wanted to convey. It would have been appreciated to make the main points on the BAR domain proteins stronger throughput, so that the reader can also follow better the choice of the models.

RESPONSE: We thank this reviewer for the advice, and hope that we have achieved this improvement with our extensive revision of the text.

Second decision letter

MS ID#: JOCES/2020/249524

MS TITLE: Endophilin-A2-dependent tubular endocytosis promotes plasma membrane repair and parasite invasion

AUTHORS: Matthias Corrotte, Mark Cerasoli, Fernando Y Maeda, and Norma W. Andrews
ARTICLE TYPE: Research Article

I am happy to tell you that your manuscript has been accepted for publication in Journal of Cell Science, pending standard ethics checks.

As you will see, one reviewer suggested minor changes that could be made to improve clarity that you may want to consider.

Reviewer 1

Advance summary and potential significance to field

In this manuscript, M Corrotte et al., report on the contribution of the bar domain containing-endophilin (specifically the ubiquitous endophilin 2) to the uptake of membrane wounds caused in fibroblasts either by the cholesterol dependent pore-forming protein SLO or the invading protozoan Trypanosoma cruzi. The authors have previously shown that several cells including epithelial cells, fibroblasts and the injury prone muscle cells rely on the calveolae endocytosis to promote resealing of the plasma membrane at the site of SLO-triggered lesions and post membrane patch delivery through lysosome exocytosis. They now report that in fibroblasts derived from mouse homozygous for a disruption of the caveolin1 gene, and therefore lacking calveolae, an endophilin 2-dependent tubular endocytosis can also work as a suboptimal process to seal and repair the wound sites at the plasma membrane. By monitoring the cholera toxin internalization in SLO-exposed cells using quantitative EM analysis, the authors were able to establish the up regulation of the tubular endocytosis upon loss of caveolae whereas by silencing the endophilin-2 expression, they showed the contribution of endophilin-2-controlled tubular endocytosis in the repair of distinct membrane wounds. Two types of wounds at the fibroblast plasma membrane were analyzed, first the SLO toxin-driven pores (a classical model) or the membrane lesion caused at the site entry by the larger trypomastigote of the T. cruzi protozoan parasite.
The context and the rationale of the work are clearly presented including the elegant use of caveolin KO cells, although it might be slightly clarified for the working hypothesis (see below the specific comments). The experimental plan is well executed with appropriate approaches including quantitative FACS analysis and qEM imaging as well confocal imaging. The data convincingly document that silencing endophilin2 in caveolae-deficient MEF cells significantly increases the membrane wound extent (i.e. decreases the resealing capacity as readily assessed by influx of

© 2020. Published by The Company of Biologists under the terms of the Creative Commons Attribution License (https://creativecommons.org/licenses/by/4.0/).

propidium iodide in a statistically relevant sample of cells). If the use of the SLO system is well introduced, the added value of the T. cruzi invading trypomastigote as a living probe for membrane repair could be more emphasized. Another issue that could be discussed in more depth concerns the still complex conclusions about the endophilin2 involvement in the two distinct membrane-resealing processes analyzed here.

Comments for the author

Corrotte et al. have appropriately addressed my concerns and even added new datasets in the revised version of their manuscript. Therefore I am pleased to now strongly recommend this well-performed study for publication in Journal of Cell Science.

Reviewer 2

Advance summary and potential significance to field

This study uncovers the interplay between caveolin-1 and endophilin-A2 in the regulation of non clathrin dependent endocytosis and plasma membrane repair, which support cellular invasion by T. cruzi.

Comments for the author

The authors have addressed all my comments. I support publication.

© 2020. Published by The Company of Biologists under the terms of the Creative Commons Attribution License (https://creativecommons.org/licenses/by/4.0/).
